# N-acetyl-l-cysteine ethyl ester (NACET) induces the transcription factor NRF2 and prevents retinal aging and diabetic retinopathy

**DOI:** 10.1016/j.redox.2025.103914

**Published:** 2025-11-03

**Authors:** Giulia Realini, Rosario Amato, Mahdi Rasa, Roberto Ceccatelli, Angela Cannavale, Laura Bottoni, Federico Marchini, Alberto Minetti, Daniela Giustarini, Alessio Canovai, Alberto Melecchi, Ines Elia, Anna Krepelova, Francesco Annunziata, Maurizio Cammalleri, Ranieri Rossi, Gian Marco Tosi, Maurizio Orlandini, Mario Chiariello, Francesco Neri, Massimo Dal Monte, Federico Galvagni

**Affiliations:** a Department of Biotechnology, Chemistry and Pharmacy, University of Siena, Via Aldo Moro 2, Siena, 53100, Italy; b Department of Biology, University of Pisa, Pisa, 56127, Italy; c Leibniz Institute on Aging – Fritz Lipmann Institute (FLI), Jena, Germany; d Istituto di Fisiologia Clinica (IFC), Consiglio Nazionale Delle Ricerche (CNR) and Core Research Laboratory, Istituto per lo Studio, la Prevenzione e la Rete Oncologica (ISPRO), Siena, 53100, Italy; e Department of Life Sciences and Systems Biology, University of Turin, Torino, Italy; f Department of Medicine, Surgery and Neuroscience, University of Siena, Siena, 53100, Italy; g Molecular Biotechnology Center, University of Turin, Torino, Italy

**Keywords:** Age-related macular degeneration (AMD), Diabetic retinopathy (DR), Oxidative stress, NF-E2-related factor 2 (NRF2), Kelch-like ECH-Associated protein 1 (KEAP1), N-Acetylcysteine ethyl ester (NACET), Cysteine (Cys), Cystine (Cyss)

## Abstract

Age-related macular degeneration (AMD) and diabetic retinopathy (DR) are leading causes of visual impairment in older people, with oxidative stress playing a central role in the development of these diseases. In this study, we showed that N-acetylcysteine ethyl ester (NACET) not only increases intracellular cysteine and glutathione levels, but also strongly stimulates the expression and activity of the transcription factor NRF2, a master regulator of oxidative stress response, in RPE cells. Using RNA interference, mass spectrometry and mutagenesis of the NRF2 regulator KEAP1, we identified direct cysteinylation of the sensor residues Cys226 and Cys613 on KEAP1 as the molecular mechanism underlying NRF2 activation after NACET treatment. Furthermore, we demonstrated that oral administration of NACET induces NRF2 activity in the retina *in vivo*, attenuates retinal aging hallmarks, and prevents diabetes-induced retinal neurodegeneration in mouse models. These results position NACET as a promising therapeutic candidate for age- and oxidative stress-related retinal diseases such as AMD and DR.

## Highlights

• N-acetylcysteine ethyl ester (NACET) boosts cysteine, glutathione and activates NRF2 in RPE cells.
• Cysteinylation of KEAP1 Cys226/Cys613 drives NRF2 activation by NACET.
• Oral NACET activates NRF2 signaling in the retina *in vivo*.
• NACET reduces retinal aging markers and diabetic neurodegeneration.
• NACET is a promising therapeutic candidate for oxidative stress-related diseases.

## Graphical abstract

**Figure:**
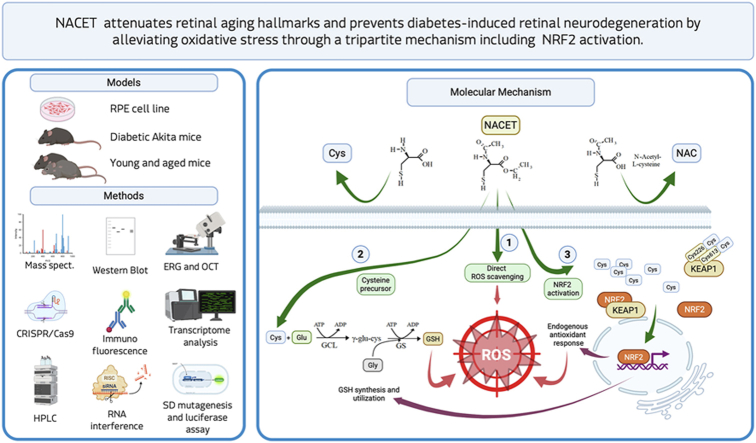

## 1 Introduction

Age-related macular degeneration (AMD) and diabetic retinopathy (DR) are among the leading causes of visual impairment and blindness in older people, and there is an unmet medical need for prevention and treatment [1]. Oxidative stress, an imbalance between reactive oxygen species (ROS) and antioxidant defense, plays an important role in the development and progression of AMD and DR, although these two diseases differ in terms of the causes of oxidative stress and the cell types predominantly affected. In AMD, the retina, particularly the macula, is highly susceptible to oxidative stress due to its high metabolic activity and constant exposure to light. With increasing age, the antioxidant defenses become weaker, which leads to increased oxidative damage to the retinal cells, especially the retinal pigment epithelium (RPE) cells. This damage contributes to RPE dysfunction, degeneration and potentially choroidal neovascularization, all of which can impair photoreceptor function and lead to vision loss [2,3]. Oxidative stress is also involved in the pathophysiology of DR. Indeed, hyperglycemia activates several pathways, including the polyol, protein kinase C, and hexosamine pathways, leading to the formation of ROS, which in turn cause dysfunction of the mitochondrial respiratory chain by directly damaging its protein components, thus triggering a vicious cycle of ROS generation [[4], [5], [6], [7]]. Finally, oxidative stress impairs microcirculation and triggers neurodegeneration, particularly affecting the survival and activity of retinal ganglion cells (RGCs) [8].

The glutathione system is a major endogenous intracellular means of protection against oxidative stress. Reduced glutathione (GSH) is a tripeptide composed of cysteine (Cys), glycine, and glutamic acid, with Cys being the rate-limiting precursor. GSH acts as a direct scavenger of ROS and as a cofactor for the enzymatic removal of ROS [9] and plays a major role in protecting RPE cells from oxidative stress [[10], [11], [12]]. In the diabetic context, the level of GSH is decreased in the retinas of sick rats and mice [13,14]. Moreover, the transcription of the catalytic subunit of glutamate–cysteine ligase, the key enzyme in GSH synthesis, is inhibited in the diabetic rat retina due to epigenetic changes in the promoter which obstacle the activity of the transcription factor NF-E2-related factor 2 (NRF2). To this regard, it is important to highlight that the improvement of glycemic control fails to rescue the prediabetic epigenetic state and NRF2 binding activity [15].

NRF2 is a master regulator of the oxidative stress response which is tightly regulated by a post-translational mechanism. Under basal conditions, Kelch-like ECH-associated protein 1 (KEAP1), the main intracellular regulator of NRF2, homodimerizes and constitutively interacts with NRF2, ultimately inducing its degradation. Indeed, KEAP1 also binds E3 ubiquitin ligase Cullin-3 to form a complex that ubiquitinates NRF2 and promotes its proteasome-mediated degradation. However, when KEAP1 is exposed to oxidative stress or xenobiotic compounds, its reactive cysteine residues are covalently modified, preventing its interaction with NRF2, which in turn leads to stabilization of NRF2, nuclear translocation, and binding to the antioxidant response elements (AREs) of hundreds of target genes. These target genes are then involved in various processes such as the antioxidant response, GSH synthesis and utilization, cellular metabolism and inflammation [16]. All in all, these observations suggest that the GSH system and NRF2 activity are promising targets for the treatment of AMD and DR. Since Cys is the limiting substrate in GSH synthesis, the preferred way to increase GSH levels in cells is to supply them with Cys or a source of this molecule. Several cysteine derivatives have been proposed for this purpose. Among them, N-acetyl-l-cysteine (NAC) is the most commonly used, although it has low lipophilicity and bioavailability. However, its esterification to NAC ethyl ester (NACET) greatly increases its ability to cross the plasma membrane and enter the cytoplasm. There it is deesterified to the more hydrophilic NAC, trapped in the cell in this form and slowly converted to cysteine. Consequently, NACET has been shown to be much more effective than NAC in increasing intracellular cysteine and GSH levels in RPE cells and improving their viability after oxidative stress [12,17]. In addition, NACET increases GSH levels in the eyes of rats and restores redox balance and DUB activity in the aging brain of mice after oral administration, demonstrating its ability to cross the blood-retinal barrier and the similar blood-brain barrier [12]. Therefore, the use of NACET for the treatment of oxidative stress-induced retinal diseases is promising. Here, we present evidence that NACET rapidly and strongly induces NRF2 expression and activity in RPE cells with a peculiar effect on transcriptome. By RNA interference, mass spectrometry analysis and targeted KEAP1 mutation, we identified direct cysteinylation of sensor Cys residues 226 and 613 of KEAP1 as the molecular mechanism underlying NRF2 activation. Furthermore, we were able to demonstrate the rescuing effect of NACET on aging and the diabetic phenotype in the retina *in vivo*.

## 2 Results

### 2.1 NACET induces NRF2 pathway in RPE cells

**Fig. 1:**
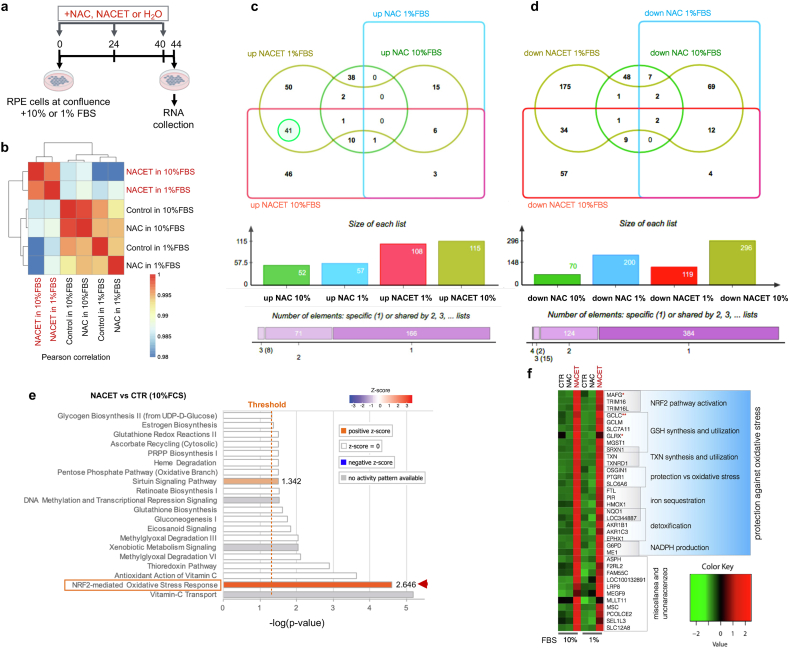
*NACET induces NRF2 pathway in RPE cells.***a**, Treatment schedule with NAC or NACET. Confluent ARPE-19 cells were cultured for 44 h in DMEM/F12 with 1 % or 10 % FBS and treated chronically with 0.4 mM NAC, NACET or H_2_O at the indicated time points. Total mRNA was extracted and RNA-seq analysis was performed. **b**, Pearson's correlation matrix visualizing the correlation values between samples. Scale bar represents the range of the correlation coefficients (R) displayed. **c**, Euler diagram showing the number of down-regulated DEGs vs untreated sample and the overlap of DEGs among the samples. **d**, As in (**c**) for up-regulated genes. A Fragments Per Kilobase of transcript per Million (FPKM) cut-off ≥1 in treated samples was used to select up-regulated DEGs and a FPKM cut-off ≥1 in untreated samples was used to select down-regulated DEGs. ∣Log2 fold change ∣≥0.6 (treated vs control) and P adj ≤0.05 were used to define DEGs. **e**, Ingenuity Pathway Analysis (IPA) of DEGs for NACET-treated vs control in 10 % FBS condition. The values of positive z-scores are reported. **f**, Heatmap of the 32 out of 41 genes serum-independently upregulated only by NACET and direct transcriptional targets of NRF2. Direct target genes of NRF2 that were upregulated by NACET only in 10 % or 1 % FBS condition are indicated by one (∗) or two asterisks (∗∗), respectively. The known function of groups of genes is indicated.

We have previously shown that NACET increases the resistance of RPE cells to oxidative stress [12]. To investigate whether the effect of NACET in preventing oxidative stress in RPE might be also related to the regulation of gene transcription, we performed a transcriptome analysis of ARPE-19 cells, an immortalized human RPE cell line, after treatment with NAC or NACET. To avoid possible bias due to culture conditions, we treated the cells under both low and high serum conditions (Fig. 1a). NACET induced a more consistent and distinct change in transcriptional profile compared to NAC (GEO dataset GSE299875, Fig. 1b–d and Supplementary Dataset 1). Ingenuity Pathway Analysis (IPA) of differentially expressed genes (DEGs) confirmed this observation and indicated that NACET specifically and strongly induced the NRF2 transcription factor pathway under both serum conditions (Fig. 1e, Supplementary Dataset 2 and Supplementary Fig. 1). Also, it is worth noting that 41 DEGs were upregulated by NACET in a serum-independent manner (Figs. 1d) and 32 of them are already described as direct NRF2 targets (Fig. 1f). Since NRF2 activation by NACET was serum-independent, all subsequent experiments were performed in 10 % FBS.

### 2.2 NACET induces NRF2 expression, nuclear localization and transcription of NRF2 target genes

**Fig. 2:**
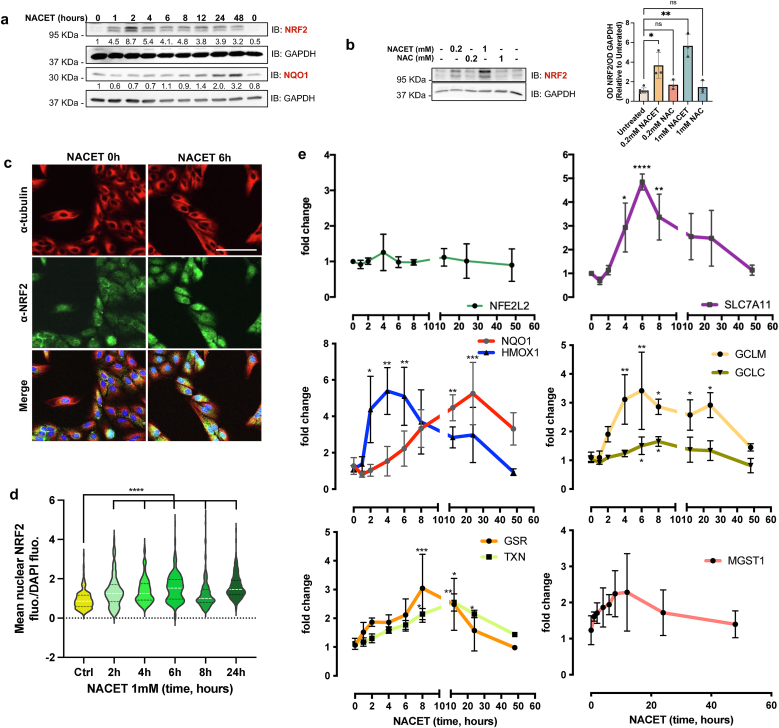
*NACET induces NRF2 expression, nuclear localization and transcription of NRF2 target genes.***a**, NACET induces the expression of NRF2 and its target gene, NQO1, at the protein level. Confluent ARPE-19 cells were treated with 1 mM NACET for the indicated times, and whole cell lysates were analyzed by Western blot with anti-NRF2 and anti-NQO1 antibodies. Anti-GAPDH immunodetection was used as loading control. **b**, NACET, but not NAC, induces NRF2 expression. Confluent ARPE-19 cells were treated for 2 h with NACET or NAC and analyzed as in (**a**) Densitometric analysis of three independent experiment is shown. Differences with control group (Untreated) were tested using Kruskal-Wallis test followed by Dunnett's multiple comparisons test. ∗∗p < 0.01, ∗p < 0.05. **c**, Immunofluorescence staining for α-tubulin and NRF2 of ARPE-19 cells treated as in (**a**) The cyan signal in Merge panels represents DAPI staining. Bar: 100 μm **d**, Fold change of fluorescence intensity of nuclear NRF2 signal after NACET treatments. Data are represented as a violin plot; horizontal lines are median (white) and interquartile range (black). Differences with control group (time 0) were tested using Kruskal-Wallis test followed by Dunnett's multiple comparisons test. N = 3, at least 45 nuclei were scored for each condition and experiment. ∗∗∗∗p < 0.0001. **e**, RT-qPCR analysis of mRNA expression of NRF2 and NRF2 target genes in ARPE-19 cells treated as in (**a**). The mRNA levels were normalized to the expression of GAPDH as an internal control. Results are represented as means ± SD of ≥3 independent experiments. Differences with control group (time 0) groups were tested using Kruskal-Wallis test or one-way ANOVA followed by Dunnett's multiple comparisons test. ∗p < 0.05, ∗∗p < 0.01, ∗∗∗p < 0.001 and ∗∗∗∗p < 0.0001.

To investigate whether NACET induces NRF2 expression at the protein level, we performed a time course analysis on confluent ARPE-19 cells treated with NACET. NACET induced a strong and rapid increase in NRF2 expression (Fig. 2a), which is not achieved by the same amount of NAC (Fig. 2b). Furthermore, immunofluorescence analysis showed that NACET leads to an increase in NRF2 protein localization in the nucleus (Fig. 2c and d and Supplementary Fig. 2). As expected based on the described activation mechanism of NRF2, mRNA level of Nuclear Factor Erythroid 2 Like 2 (NFE2L2), the gene encoding NRF2, showed no significant changes, suggesting that also the NACET-mediated induction of NRF2 expression depends on a post-transcriptional mechanism (Fig. 2e). Conversely, NACET strongly induced mRNA expression of several of NRF2 target genes (NQO1, HMOX1, GSR, TXN, SLC7A11, GCLM, GCLC, and MGST1), confirming the transcriptome results (Fig. 2e).

**Fig. 3:**
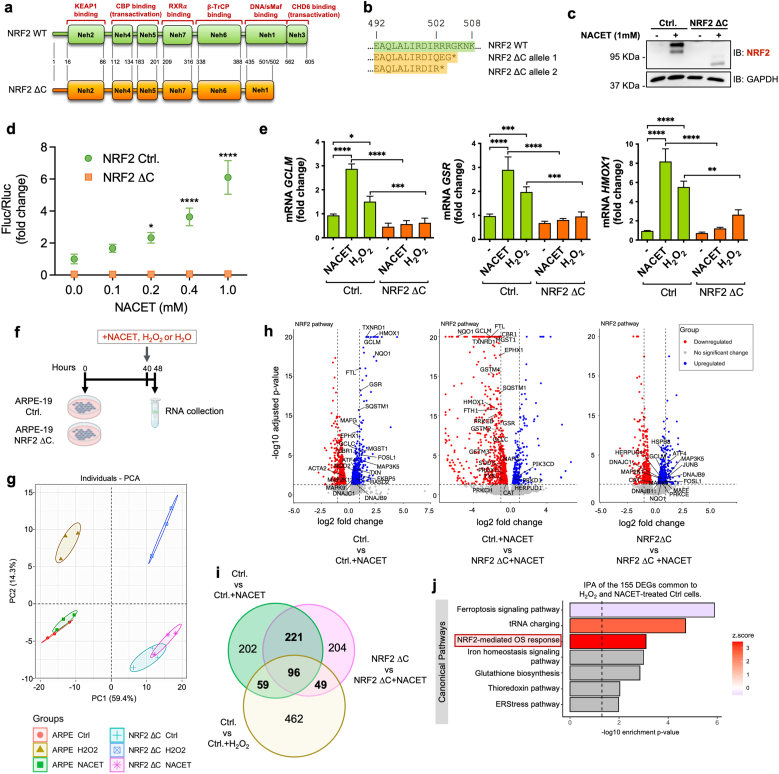
*NACET induces NRF2 activity.***a**, Schematic view of wild type and CRIPR/Cas9 edited NRF2 protein (NRF2 ΔC). The functional domains and the site of mutation inserted by CRISPR/Cas9 are indicated. The numbers of the amino acid residues are numbered from N-terminal methionine. **b**, C-terminal amino acid sequences of the NRF2 alleles obtained by genome editing. **c**, Western blot analysis showing the expression of NRF2 and, as loading control, GAPDH, in control and NRF2-edited ARPE-19 cells treated or untreated with 1 mM NACET for 4 h. Control cell line (Ctrl.) was obtained transducing ARPE-19 cells with the pLCv2-derived lentiviral particle without sgRNA. **d**, Induction of NRF2 activity by NACET was tested by transfection of Ctrl and NRF2ΔC cells with the pGL4.37 (lucP/ARE/Hygro) vector and treatment with NACET at the indicated concentrations for 24 h. The data are represented as the ratio of Firefly to Renilla luciferase activity (Fluc/Rluc). N = 3. Differences with the control group (0 mM NACET) were tested using two-way ANOVA followed by Dunnett's multiple comparisons test. **e**, Graphs showing the RT-qPCR quantification of HMOX1, GCLM and GSR mRNA after 8 h of induction with 1 mM NACET or 0.2 mM H_2_O_2_. H_2_O_2_, a well-known NRF2 activator, was used as positive control. Data are normalized versus GAPDH expression and results are shown as fold induction compared to untreated control ARPE-19 cells. Differences between groups were tested using two ANOVA followed by Tukey's multiple comparisons test. N = 2. ns, not significant. **f**, Treatment schedule with 1 mM NACET or 0.2 mM H_2_O_2_. 20 × 10^3^ cells/cm^2^ were seeded and 40 h later treated as indicated. N = 3. **g**, PCA of the transcriptome data. **h**, Volcano plots of the DEGs obtained by the comparison of the indicated datasets. Dots corresponding to genes of the NRF2 signalling pathway are named in the plots. **i**, Venn diagram reporting the number of DEGs in the three conditions indicated. **j**, IPA of the 155 DEGs common to H_2_O_2_- and NACET-treated control cells compared to untreated control cells.

To unequivocally demonstrate that NRF2 is responsible for NACET-dependent transcriptional activation of the upregulated genes identified by RNA-seq, we used CRISPR/Cas9 technology to generate an ARPE-19 cell line expressing a non-functional form of NRF2 (NRF2 ΔC) because the C-terminal portion, which contains approximately half of the DNA/sMaf binding domain and the entire CHD6 binding domain, was removed (Fig. 3a–c) [18]. To demonstrate the capability of NACET to promote transcriptional activation of target genes through NRF2-ARE binding, we transfected NRF2 ΔC and control ARPE-19 cells with a construct carrying the luciferase gene under the control of an artificial ARE-containing promoter and subsequently performed a luciferase assay. As shown in Fig. 3d, we observed an induction of luciferase activity directly proportional to NACET concentration in control cells, but not in NRF2 ΔC expressing cells. The same effect is observed on the activation of expression of the endogenous classical NRF2 target genes GCLM, GSR and HMOX1 (Fig. 3e).

To further confirm the activation of the NRF2 pathway by NACET and to evaluate whether this activation is peculiar of NACET treatment or the effect of an indirect oxidative stress, we performed a transcriptome analysis on ARPE-19 and NRF2 ΔC cells following short (8 h) NACET or H_2_O_2_ treatments (GEO dataset GSE299876). Firstly, to determinate the relationship between all samples, we conducted the Principal Component Analysis (PCA) of the transcriptome data. The first principal component (PC1) accounted for 59.4 % of the expression variance and separated control from NRF2 ΔC ARPE-19 cells, indicating that the presence/absence of a functional NRF2 explains the variance in this set of samples. The second principal component (PC2) accounted for 14.3 % of the variance in expression and segregated the samples as a result of the different stimuli, with H_2_O_2_ having a greater effect on the transcriptome than NACET treatment (Fig. 3f and g). Volcano plots of DEG distributions showed that NRF2 target genes were upregulated by NACET in control cells but not in NRF2 ΔC cells, confirming the role of NRF2 in their regulation by NACET (Fig. 3h and Supplementary Dataset 3). Analysis of DEGs in control cells treated with NACET or H_2_O_2_ and in NRF2 ΔC cells treated with NACET showed that the modulation of 261 (45 %) DEGs in NACET-treated control cells was NRF2-dependent, and of these, 59 matched the list of DEGs in H_2_O_2_-treated control cells (Fig. 3i and Supplementary Dataset 4). Moreover, the IPA of DEGs common to H_2_O_2_- and NACET-treated control cells mainly showed upregulation of the NRF2 signaling pathway and downregulation of ferroptosis, which is normally inversely regulated by NRF2 (Fig. 3j). In summary, these data confirm that H_2_O_2_ and NACET treatments are two distinct stimuli that mainly share the activation of the NRF2 signalling pathway.

### 2.3 Molecular mechanism of NACET-regulated NRF2 expression

**Fig. 4:**
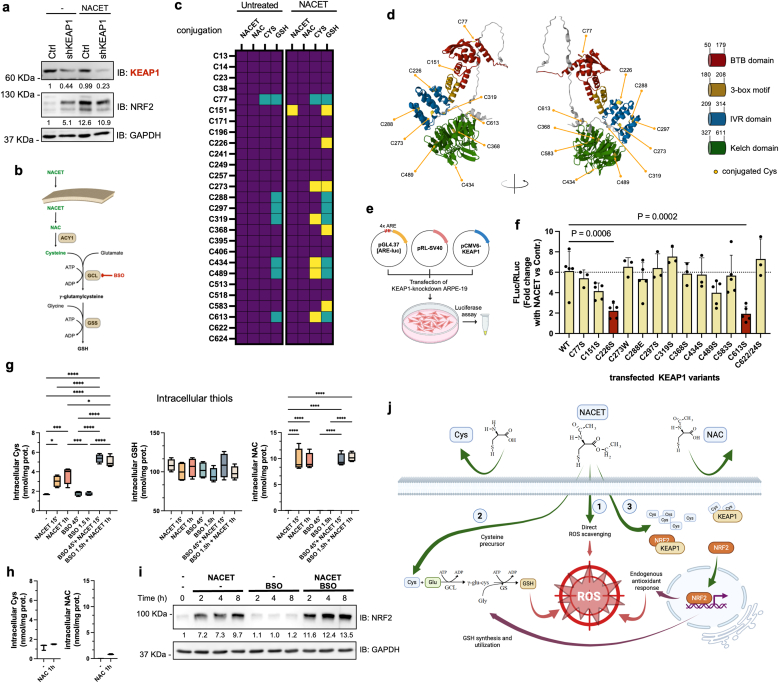
*NACET increases intracellular levels of Cys, which directly conjugate to Cys residues 226 and 613 of KEAP1 and induce NRF2 expression.***a**, Western blot analysis of KEAP1 and NRF2 expression in ARPE-19 cells following lentiviral-mediated expression of shRNAs targeting 3′UTR of KEAP1 mRNA (shKEAP1) or GFP (Ctrl). Where indicated. Cells were treated with 1 mM NACET for 2 h **b**, Schematic representation of the GSH synthesis pathway. The enzymes involved are boxed; abbreviations: ACY1, aminoacylase 1; GCL, glutamate-cysteine ligase; GSS, glutathione synthetase. **c**, Heatmap showing the status of sensor cysteine residues identified by mass spectrometry analysis of purified 3xFLAG-KEAP1 transfected into HEK 293 cells treated and not treated with NACET: unconjugated (purple squares), NACET-, NAC-, Cys- or GSH-conjugated in NACET-treated and untreated samples (cyan squares) or only in NACET-treated samples (yellow squares). **d**, AlphaFold model showing the domains of KEAP1 and the relative position of the 12 Cys residues found conjugated in the MS analysis. **e**, Scheme of the transfection protocol for the analysis of NRF2 transactivation activity. pRL-SV40 was used for the constitutive expression of Renilla Luciferase for normalization. **f**, NRF2 activity measured as transactivation of the ARE-luc reporter gene in cells silenced for endogenous KEAP1 expression. KEAP1 silenced cells were transfected with reporter plasmids and one plasmid for the expression of shRNA-resistant KEAP1 (wt or mutant, as indicated). 8 h post transfection, the cells were grown for 24 h in growth medium ±0.2 mM NACET. KEAP1 Cys residues were substituted with serine, glutamic acid (C288E) or tryptophan (C273W) as described by Suzuki et al. [15]. Values represent the fold change in normalized luciferase activity in cells transfected for the expression of the corresponding KEAP1 variant and are expressed as means ± SD (N = 3–5). Differences were tested using one-way ANOVA followed by Dunnett's multiple comparisons test. **g,** Confluent ARPE-19 cells were pre-treated for 30 min with 100 μM BSO where indicated and then incubated with 0.2 mM NACET for the indicated time points, lysed by TCA-EDTA solution and the intracellular thiols were measured by HPLC. Thiol concentrations are normalized for the total protein content and expressed in nmol/mg protein. Measurements are the means ± SD of 4 independent experiments. Differences were tested using one-way ANOVA followed by Tukey's multiple comparisons test. ∗p < 0.05, ∗∗∗p < 0.001, ∗∗∗∗p < 0.0001. For clarity, not all the significant differences are indicated. **h**, Confluent ARPE-19 cells were incubated with 1 mM NAC for 1 h, and thiols were extracted and measured as in (**g**). **i**, Whole lysates of cells treated as in (**g**) were analyzed by Western blot with anti-NRF2 antibody. Anti-GAPDH antibody was used as a loading control. Densitometric analysis of bands is shown. **j**, Cartoon showing the proposed NACET's tripartite mechanism of action.

Next, we investigated the molecular mechanism by which NACET regulates NRF2 expression. Proteasomal-dependent post-transcriptional degradation of NRF2 is the major negative regulatory mechanism for its activity, and the E3 ligase KEAP1 is the most important and best characterized driver of this process [19]. To investigate the role of KEAP1 in NACET-dependent expression, we transduced ARPE-19 cells with lentiviral particles for the expression of shRNAs targeting the 3′UTR of KEAP1 mRNA or GFP (as a negative control). Knockdown of KEAP1 resulted in increased expression of NRF2, similar to that observed after treatment of control cells with NACET (Fig. 4a). This observation and the lack of effect of NACET on NFE2L2 transcription (Fig. 2e) suggest that NACET induces NRF2 at least in part by inactivating KEAP1. To test this hypothesis and to identify the sensor Cys residues of KEAP1 involved in the response to NACET, we transfected a plasmid for the expression of a 3xFLAG-tagged KEAP1 into HEK-293 cells, which were then treated with or without NACET. The tagged KEAP1 was purified by affinity and analyzed by mass spectrometry (MS). NACET can freely cross the plasma membrane and is rapidly deesterified within the cell to NAC, which in turn is converted to Cys. The resulting higher availability of Cys leads to an increase in GSH synthesis (Fig. 4b) [12]. Since each of these four molecules has a reactive thiol group that can react with sensor Cys residues of KEAP1, we analyzed all of these possible KEAP1 conjugations in NACET-treated cells by MS and identified Cys residue 151 as conjugated to NACET, Cys residues 151, 226, 273, 368 and 583 as conjugated to GSH, Cys residues 273, 319, 434, 489 and 613 as conjugated to Cys and no residues as conjugated to NAC (Fig. 4c and d). To determine which of these residues actually regulate KEAP1 activity after treatment with NACET, we cotransfected the KEAP1-silenced cells with plasmids for ARE-driven expression of the luciferase reporter gene and for constitutive expression of the KEAP1 ORF (wt or point mutated in the identified sensor Cys residues) (Fig. 4e). Cys residues 622 and 624 were also included in this analysis, although these Cys residues were not recognized as conjugated in the MS analysis, as they have been described to be involved in KEAP1 inactivation in synergy with residues 226 and 613 [19]. The transfected wild-type and all mutant forms of KEAP1 strongly repressed NRF2 activity compared to GFP-transfected samples, suggesting they are correctly expressed and active (Supplementary Fig. 3). The analysis revealed that only mutations of residues 226 and 613 resulted in a significantly defective induction of ARE activity in the presence of NACET (Fig. 4f). Since KEAP1 was directly cysteinylated *in vitro* by free Cys at residues 226 and 613 [20], and NACET but not NAC induced an increase in the intracellular concentration of Cys, but not of GSH, in the short time period (Fig. 4g and h), we wondered whether GSH conjugation of KEAP1 was the functional event or only a secondary consequence of the previous cysteinylation. To investigate this point, we treated the RPE cells with BSO (the irreversible inhibitor of GCL) for 30 min before treating them with NACET for 15 min or 1 h to avoid the consumption of Cys in favor of GSH synthesis. Under these conditions, we observed a more sustained increase in intracellular Cys levels by NACET after BSO treatment (Fig. 4g) and the increased expression of NRF2 (Fig. 4i), suggesting that KEAP1 is inactivated by direct cysteinylation of both residues 226 and 613 after treatment with NACET, similar to what has been observed *in vitro* [20]. In summary, thanks to its lipophilicity and independent of a specific transporter, NACET enters the cells where it can alleviate oxidative stress through a tripartite mechanism: 1) it directly scavenges ROS, 2) it supplies the cell with Cys, the limiting precursor of GSH, and 3) it induces the activation of NRF2 (Fig. 4j).

### 2.4 NACET treatment partially rescues the transcriptional phenotype of the aging retina

**Fig. 5:**
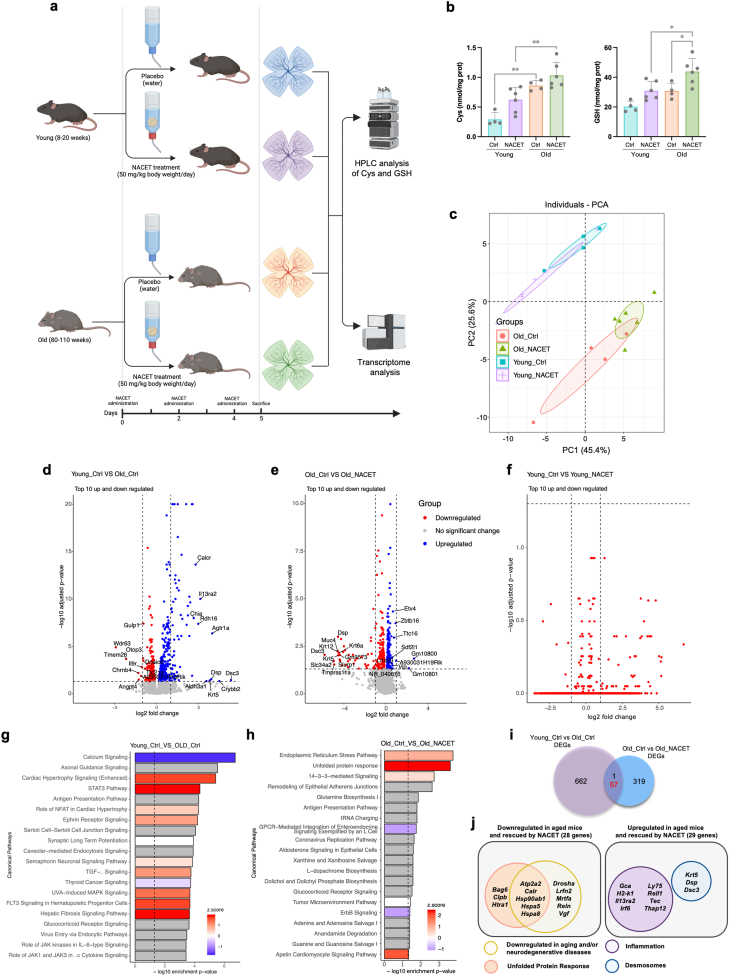
*NACET treatment partially rescues the transcriptional phenotype of aging retina*. **a**, Scheme of the treatment with NACET of young (8–20 weeks) and old (80–110 weeks) C57BL6/J mice. **b**, Cys and GSH levels in the retinas. Thiol concentrations are normalized for the total protein content and expressed in nmol/mg protein. Control groups: 4 mice/group, NACET-treated groups: 6 mice/group. Measurements are the means ± SD. Differences between groups were tested using two-way ANOVA followed by Šídák's multiple comparisons test. ∗p < 0.05, ∗∗p < 0.01. **c**, PCA of normalized RNA-seq data. **d**, Volcano plot shows that there are 720 DEGs between the young ctrl and the old ctrl, and the number of upregulated and downregulated genes in the old ctrl are 410 and 310, respectively. **e**, Volcano plot of young ctrl VS young NACET shows no significant changes between the two groups, while **f**, Volcano plot of old ctrl VS old NACET shows that there are 377 DEGs and the number of upregulated and downregulated genes in old NACET-treated mice are 185 and 192, respectively. The group named before “VS” is the base group. **g**, IPA of the DEGs between young and old mice. **h,** IPA of the DEGs in the retinas between untreated and NACET-treated old mice. **i**, Venn diagram showing the overlapping DEGs between the indicated analyses. The red number indicates the opposite regulation between the two analyses. **j**, Venn diagrams showing the functional analysis of the 57 DEGs in aging that were rescued by NACET. Only the names of the genes associated with the major functional groups identified are indicated. Areas are proportional to the number of genes.

Chronic oxidative stress is widely recognized as a key factor in retinal cell damage and death in retinal degenerative diseases and activation of NRF2 is emerging as an attractive therapeutic approach to counteract this stress and prevent AMD and DR. With the aim of proposing NACET as a therapeutic and preventive agent against AMD, we investigated the effects of NACET *in vivo* on the aging retina of mice. To this end, we treated young (8–20 weeks) and old (80–110 weeks) C57BL/6J mice with NACET added to drinking water (Fig. 5a). After 5 days of treatment, we measured Cys and GSH concentrations in the retinas by HPLC analysis and found that both were significantly higher in the retinas of old mice compared to those of young mice, as well as in the retinas of NACET-treated mice, regardless of age (Fig. 5b). This suggests that the aged retina requires an enhanced antioxidant response and that NACET can increase the natural GSH antioxidant system of the retina. Furthermore, we performed a transcriptome analysis of young and old mouse retinas after NACET treatment (GEO dataset GSE299877). First, we analyzed the expression of markers of the major cellular component of the retina as quality control for the process of tissue dissection (Supplementary Fig. 4a) and found no significant differences in cellular representation. The differential gene expression data were then subjected to PCA (Fig. 5c). As a result, PC1 accounted for 45.4 % of the variance in expression and segregated the samples as a result of NACET treatment, demonstrating that young and old retinas respond differently to NACET treatment. PC2 accounted for 25.6 % of the variance in expression and showed separation of samples due to age-related differences that diminished in the presence of NACET treatment. When comparing the expression pattern between young and old control groups (FPKM ≥1, p adj≤0.05), we detected 720 DEGs and the number of upregulated and downregulated genes were 410 and 310, respectively. No significant changes were detected between the young control group and the young NACET-treated group, while we identified 377 DEGs in the comparison between the old control group and the old NACET-treated group, including 186 upregulated and 192 downregulated genes (Fig. 5d–f, Supplementary Dataset 5). The IPA of DEGs shows that calcium, STAT3, ephrin receptor and TGF signaling pathways were among the most enriched pathways in old compared to young retinas (Fig. 5g), while ER stress signaling pathway and unfolded protein response (UPR) are the functions associated with the highest scored network in the comparison between control and NACET-treated old mice (Fig. 5h). Interestingly, analysis of putative upstream regulators of DEGs in old compared to young retinas reported upregulation of the activity of several factors involved in inflammation and the senescence-associated secretory phenotype (SASP), such as tumor necrosis factor-α (TNF-α), interferon gamma (IFN-γ), and Toll-like receptor (TLR) (Supplementary Fig. 5a). Among the regulators identified by comparing control and NACET-treated old retinas, the activity of inflammatory players such as IL-1β, IL-13, STAT5A, G-CSF (CSF3), and transcription factor p65 (RELA) was reported to be downregulated. Importantly, the activity of NRF2 was increased in NACET-treated old mice, confirming its activation by NACET also in the retina *in vivo* (Supplementary Fig. 5b). When we focus on the age-dependent DEGs, the expression of 15 % of them (57 genes) was rescued by NACET treatment (Fig. 5i and Supplementary Table 1). When analyzing their function, we found that important genes involved in the UPR were rescued by NACET and genes involved in inflammation were instead reduced (Fig. 5j, Supplementary Fig. 4b and c and Supplementary Fig. 5c). In addition, 10 genes whose expression was restored by NACET have been already described as downmodulated in aging and/or neurodegenerative diseases (Fig. 5j and Supplementary Fig. 4c). Taken together, these data suggest that NACET can reduce inflammation, counteract the age-related decline of some homeostatic pathways, and halt natural retinal aging by both inducing NRF2 activity and increasing GSH synthesis.

### 2.5 NACET treatment rescues the retinal phenotype in diabetic mice

**Fig. 6:**
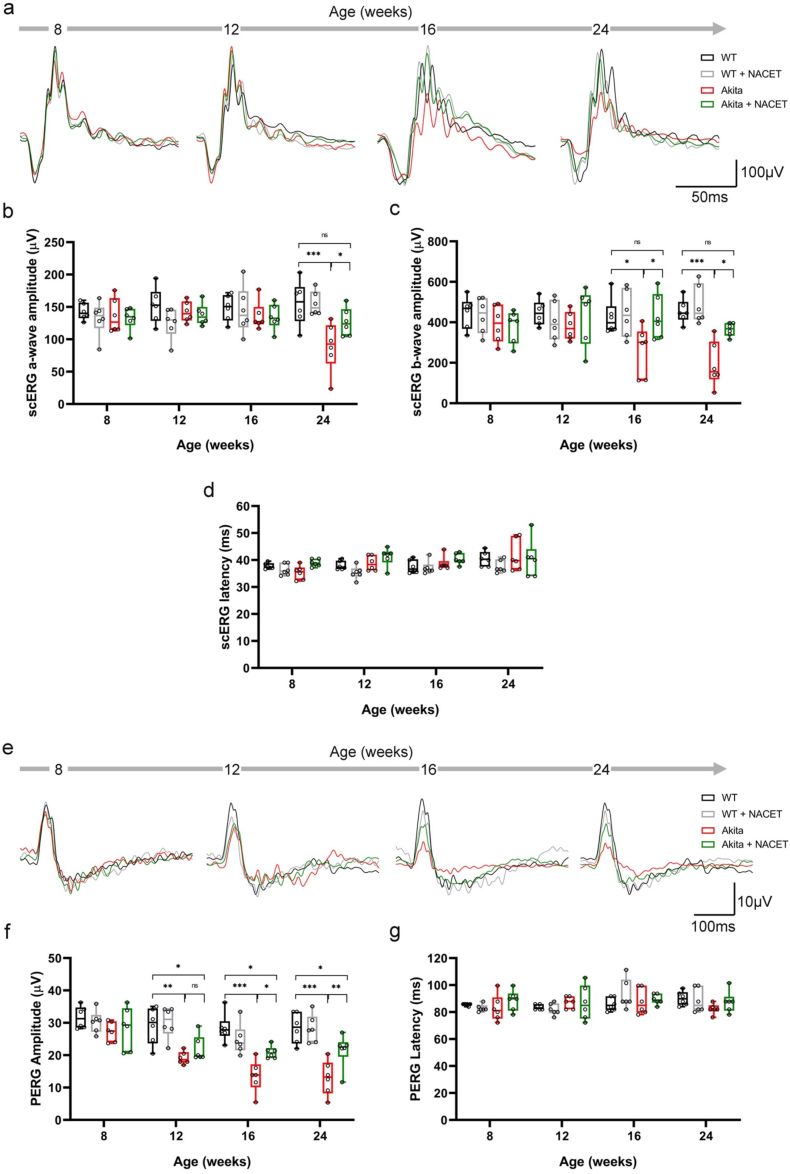
*NACET treatment rescues retinal function in diabetic mice.* Retinal activity was assessed by performing electroretinogram (ERG) recordings at 8, 12, 16 and 24 weeks of age. At each time-point, wild type (WT) or Akita mice either untreated or treated with NACET were subjected to a recording routine providing (**a-d**) the analysis of the overall retinal activity with scERG and (**e-g**) the analysis of retinal ganglion cell (RGC) activity with PERG. **a-d,** Representative scotopic ERG waveforms (**a**) and related quantification of a-wave (**b**) and b-wave (**c**) amplitudes as well as of latency (**d**) recorded at 10 cd s/m^2^ light intensity. **e-g,** Representative PERG traces (**e**) and related quantification of PERG amplitudes (**f**) and latency (**g**). Differences were tested using one-way ANOVA followed by Tukey's multiple comparisons test. ∗p < 0.05, ∗∗p < 0.01 and ∗∗∗p < 0.001.

**Fig. 7:**
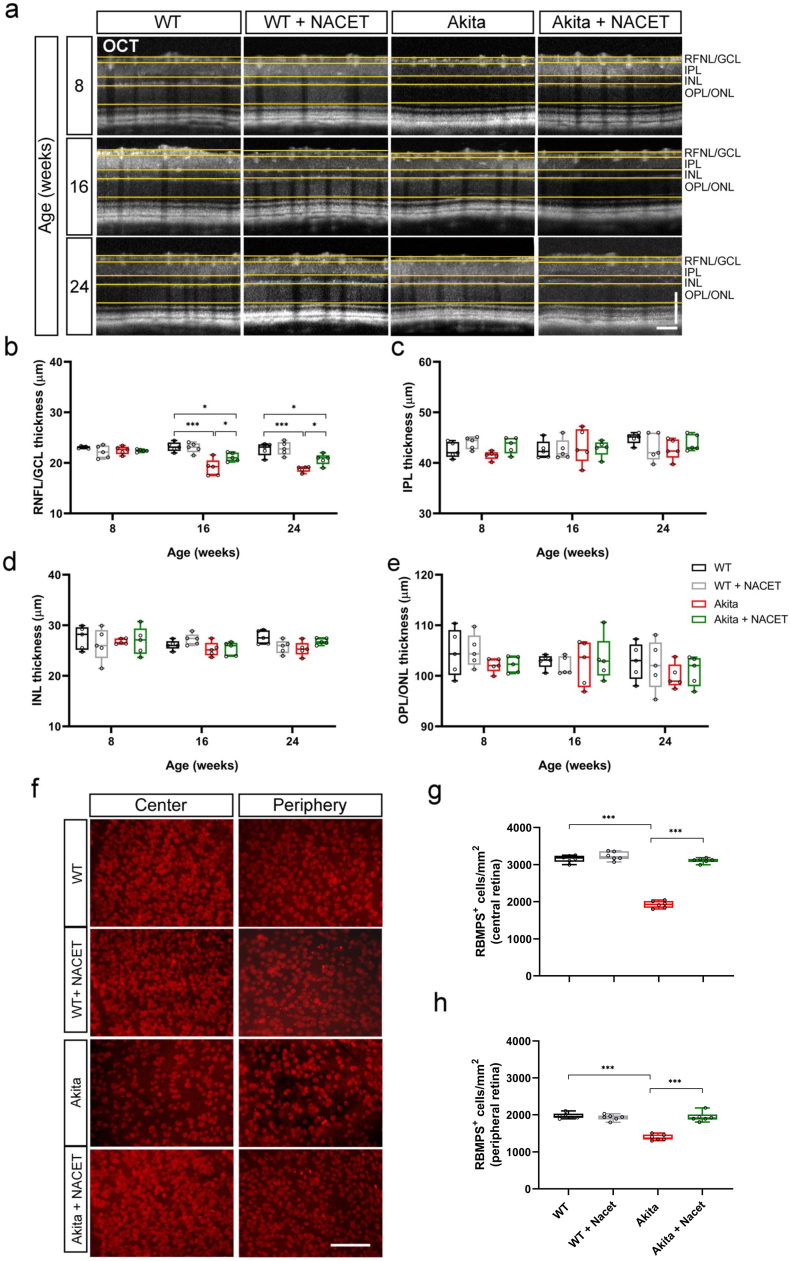
*NACET treatment preserves retinal thickness and RGC density in diabetic mice. In vivo* analysis of retinal morphology in WT and Akita mice either untreated or treated with NACET at 8,16, and 24 weeks of age was assessed by image-guided OCT (**a-e**), while RGC density was evaluated by immunohistochemistry (**f-h**). **a,** Circular B-scans were processed for segmentation of retinal layers. **b-e,** Quantitative measurements of thickness of retinal nerve fiber layer/ganglion cell layer (RNFL/GCL; **b**), inner plexiform layer (IPL; **c**), inner nuclear layer (INL; **d**), and outer plexiform layer/outer nuclear layer (OPL/ONL; **e**). Differences were tested using two-way ANOVA followed by Bonferroni's multiple comparison test. ∗p < 0.05 and ∗∗∗p < 0.001. **f,** Representative images of RBPMS staining in central and peripheral areas of retinas. Scale bar: 100 μm **g, h,** Analysis of RBPMS-positive cell density, differentially sampled from the central (**g**) and peripheral (**h**) areas of the retina. Differences were tested using one-way ANOVA followed by Tukey's multiple comparisons test. ∗∗∗p < 0.001.

Finally, we wondered whether NACET can also rescue the healthy phenotype in diabetic retinopathy. To test this hypothesis, we treated heterozygous Ins2Akita mice (hereafter referred to as Akita) with NACET from 4 weeks of age for 20 weeks. Akita mice develop insulin-dependent diabetes at 3–4 weeks of age and retinal complications at 24 weeks of age [21]. We analyzed the overall activity of the retina by electroretinogram (ERG) and observed a reduction in the amplitude of the a-wave in Akita mice at 24 weeks of age, indicating impaired photoreceptor function. The amplitude of the b-wave, which mainly reflects the light-induced activity of the ON-center bipolar and Müller cells, was also reduced in the diabetic animals, but already at the age of 16 weeks. Treatment with NACET rescued both a- and b-wave amplitudes, had no effect on glycemia, and produced only a small, though significant, rescue of body weight in AKITA mice (Supplementary Fig. 6, Supplementary Fig. 7a and Fig. 6a–c). The scotopic ERG (scERG) session was followed by pattern ERG (PERG) for the assessment of RGC function, obtaining similar results as for scERG (Supplementary Fig. 7b and Fig. 6e,f). No noteworthy changes were observed in the latency of a-wave and PERG (Fig. 6d–g). Retinal morphology was investigated by image-guided optical coherence tomography (OCT) and immunofluorescence staining of the retinas. Longitudinal analysis of retinal structure by OCT showed a significant decrease in retinal nerve fiber layer (RNFL) and ganglion cell layer (GCL) thickness, which was associated with an increase in the severity of DR in patients [22]. Even in this case, the phenotype was rescued by NACET treatment (Fig. 7a–e). To confirm the protective effect of NACET on the RGCs of the diabetic mice, we performed immunofluorescence staining of the retinas with an antibody against RNA-binding protein multiple splicing (RBPMS), which is considered a selective marker for RGCs. Akita mice showed a significant decrease in the number of RBPMS + cells in both the central and peripheral areas of the retina, and this phenotype was completely reversed by treatment with NACET (Fig. 7f–h).

**Fig. 8:**
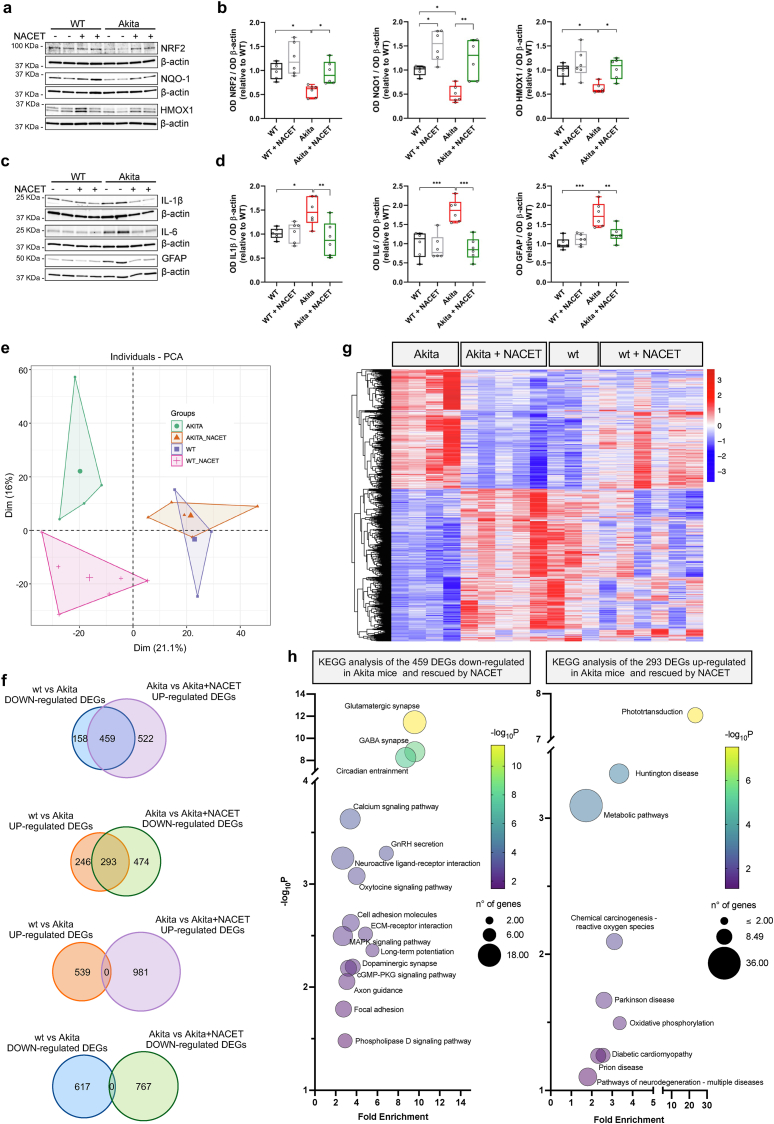
*Molecular analysis of NACET treatment in retinas of healthy and diabetic mice.* WT and Akita mice were either untreated or treated with NACET from 4 weeks of age for 20 weeks. **a,b**, Whole lysates of the retinas were analyzed by Western blot with the indicated antibodies. Anti-β-actin immunodetection was used as loading control. Representative blots are shown. **a**, NACET rescues the expression of NRF2 and its regulatory targets NQO1 and HMOX1 in the diabetic retinas at the protein level. **c**, NACET reduces the expression of IL-1 β, IL-6 and GFAP, markers of neuroinflammation and gliosis, in diabetic mice. **b,d,** Quantification of the expression of the analyzed genes as in **a** and **c**. 6 retinas from wt and 6 from Akita mice were analyzed. **e**, PCA of the transcriptome data of the retinas. Treatment of Akita mice with NACET rescues the transcriptome profile of WT mice. **f**, Venn diagram reporting the number of DEGs between the indicated groups. The group named before “vs” is the base group.∣Log2 fold change∣≥0 and P adj ≤0.05 were used to define DEGs. **g**, Heatmap showing the expression of the DEGs between Akita mice treated and untreated with NACET. **h**, Pathways identified by KEGG enrichment analysis of the 459 DEGs down-regulated in WT vs AKITA mice and up-regulated in Akita vs Akita treated with NACET and of the 293 DEGs up-regulated in WT vs Akita mice and down-regulated in Akita vs Akita treated with NACET (only context pertinent pathways with p < 0.05 and counting ≥6 genes are reported).

To evaluate whether the protective effects of NACET on retinal function and structure may correlate with improved antioxidant defences, the levels of NRF2 and some of its targets were evaluated. As shown in Fig. 8a and b, the levels of NRF2, NQO1 and HMOX1, which in Akita mice were significantly lower than in WT controls, were increased after NACET administration. The antioxidant effects of NACET were paralleled by anti-inflammatory activity, as evidenced by reduced levels of markers of both neuroinflammation and gliosis (Fig. 8c and d). Finally, to investigate the effect of NACET in diabetic retinas at the level of gene transcription, we performed a comparative transcriptome analysis (GEO dataset GSE299879). PCA shows that treatment of Akita mice with NACET restored a transcriptomic profile that overlaps with that of WT mice (Fig. 8e). Indeed, analysis of DEGs shows that NACET restored the expression of 459 (46.8 %) downregulated and 293 (38.2 %) upregulated genes in the retina of Akita mice (Supplementary Dataset 6 and Fig. 8f and g). KEGG (Kyoto Encyclopedia of Genes and Genomes) pathways rescued by NACET identified by functional annotation of DEGs are shown in Fig. 8h.

## 3 Discussion

Oxidative stress plays a major role in the development and progression of AMD and DR, and the tripeptide GSH and the transcription factor NRF2 are key players in maintaining the redox homeostasis of the cell. We have previously shown that the antioxidant NACET acts as a ROS scavenger and, in contrast to its precursor NAC, increases the intracellular level of reduced GSH in RPE cells *in vitro* and in rat eyes by supplying the cells with the intracellular limiting metabolite cysteine [12]. In this study, we have shown for the first time that NACET also strongly induces the expression and transcriptional activity of NRF2. Thus, unlike many other NRF2 inducers, this simple molecule combines most of the functions normally sought in an antioxidant molecule. Regarding the molecular mechanism of NRF2 activation, we identified Cys 226 and Cys 613 as KEAP1 residues that sense NACET treatment through their conjugation with intracellular free Cys, whose concentration is rapidly pushed up by NACET decay. Moreover, by mass spectrometry analysis, we demonstrated for the first time that these two Cys residues are cysteinylated within the cell. Although this is consistent with what has been described for NRF2 activation following overexpression of the cystine/glutamate exchange system Xc-in the mesenchymal stem-like subtype of triple-negative breast cancer cells [20], because the involvement of KEAP1 cysteinylation at residues 226 and 613 in the NACET-mediated increase of NRF2 activity is demonstrated only by transient transfection experiments, we cannot exclude the possibility that other mechanisms or KEAP1 residues are involved.

Although NACET has been considered for many years as a very interesting antioxidant molecule, it has never been studied *in vivo* in an animal model for its ability to rescue an aging or pathological phenotype [17,23]. To investigate this point for retinopathies, we used two different animal models: (i) young and aged mice treated with NACET to evaluate the effect of the drug on retinal aging as a model for events triggering the AMD phenotype, and (ii) Akita mice as a characterized and robust model for DR.

Transcriptome analysis of aged retinas confirmed the induction of NRF2 signaling *in vivo* after oral administration of NACET, while comparison between young and old mice revealed that several upstream regulators whose signaling pathways are dysregulated in aging are associated with inflammation and SASP. SASP, which results from chronic senescence, is a major source of the chronic inflammation that is typical of aging and age-related diseases such as AMD [24,25]. Interestingly, 8 of the 29 genes that are upregulated in the aged retina and whose expression is rescued by NACET are involved in inflammatory processes, such as *Il13ra2* and *Ly75*. In particular, *Il13ra2* encodes the interleukin-13 receptor subunit alpha-2 (L13Rα2), a transmembrane receptor that binds and neutralizes the anti-inflammatory cytokine IL-13 [26]. In the context of retinal degeneration, IL13Rα2 expression is induced by photosensitization of N-retinylidene-N-retinylethanolamine (A2E), which triggers telomere dysfunction and accelerates RPE senescence [27]. LY75 is a marker of immune activation that is expressed on the surface of various immune cells and in aging mice, LY75 is overexpressed in the RPE/choroid complex and retina, suggesting the involvement of inflammation and immune activation in the pathogenesis of AMD [28,29].

Three other genes (*Krt5*, *Dsp* and *Dsc3*) that are upregulated during aging and rescued by NACET encode desmosomes and hemidesmosome‐associated proteins. RPE cells release exosomes containing desmosome and hemidesmosome proteins under chronic oxidative stress. It is worth noting that KRT5 and DSP are enriched in exosomes released by iPSC‐derived RPE cells from donors at high genetic risk of AMD compared to cells from low-risk donors [30].

Among the genes downregulated in aged retinas and rescued by NACET, eight genes (*Bag6*, *Clpb*, *Htra1*, *Atp2a2*, *Calr*, *Hsp90ab1*, *Hspa5* and *Hspa8*) are involved in the unfolded protein response (UPR). Oxidative stress can trigger the accumulation of misfolded proteins in the endoplasmic reticulum (ER), leading to the onset of ER stress. To combat ER stress, cells activate the UPR signaling network. When chronically stimulated, these pathways exacerbate oxidative stress and lead to the activation of pro-apoptotic programs associated with pro-inflammatory signaling, leading to retinal degeneration and AMD [[31], [32], [33]]. However, mild UPR leads to upregulation of ER chaperones that increase the ER's ability to fold proteins and promote the degradation of misfolded proteins, thereby protecting retinal cells from cell death caused by oxidative stress [34]. From this perspective, it is noteworthy that the transcripts of *Hspa8*, *Hspa5*, *Hsp90ab1*, and *Calr* encode molecular chaperones. Interestingly, mutations in molecular chaperones have been found in inherited retinal dysfunction and degeneration [35], HSP90α deficiency causes retinal degeneration [36] and the expression of HSPA5 plays an important role in maintaining the structural integrity of cone photoreceptors and is drastically reduced in donor eyes of atrophic AMD patients [37]. In addition, CALR also has an antiangiogenic function and inhibits ocular neovascularization in rodent models of oxygen-induced retinopathy and laser-induced choroidal neovascularization [38]. Finally, a very interesting gene that is downregulated in aged retinas and rescued by NACET is *Vgf*. It encodes the protein VGF nerve growth factor inducible, which is expressed by retinal cells and protects RGCs from death caused by optic nerve crush [39,40]. However, its role in the development of AMD remains to be investigated, as does its therapeutic potential. All in all, our analysis showed for the first time that NACET can rescue the expression of genes involved in retinal aging *in vivo*. Although only 57 genes were rescued across 720 DEGs between old and young retinas, this result is promising, considering that a relatively short treatment (5 days) was performed. Longer treatments will be performed to achieve a stronger effect.

In the DR model, the hyperglycemic state triggers oxidative stress and the activation of neuroinflammation and gliosis, processes that precede microvascular damage and lead to retinal damage that ultimately results in altered retinal function [8,41]. As shown here, NACET administered at the onset of diabetes, before the development of DR, preserves retinal function and prevents retinal thinning by reducing all three processes. Our analysis of the retinal transcriptome profile confirms the effect of NACET on both oxidative stress (by rescuing pathways that are upregulated by diabetes and include genes involved in the respiratory chain, such as *Cox6a1*, *Uqcrc1*, *Ndufs2*, *Vdac2*, *Ndufv3*, *Ndufv1*) [42,43] and neurodegeneration (by rescuing pathways that are downregulated by diabetes and include genes encoding ionotropic glutamate receptor subunits, such as Gria1-4, *Grin1* and *2b*, *Grik1* and *2*, and *Grm7,* all markers for neuronal cells and RGCs in particular) [44,45] (Supplemented Dataset 7). Furthermore, we demonstrated that NACET restores the expression of NRF2 in the retina of diabetic mice. This finding is consistent with the important role of NRF2 activity in DR. Indeed, it was recently shown that NRF2 expression and activity is reduced in the streptozotocin-induced rat model of diabetic retinopathy and that inhibition of NRF2 degradation by an inhibitor of Cullin-3 neddylation alleviates DR by counteracting oxidative stress and inflammation [46]. In addition, diabetic NRF2 knockout mice showed a reduction in retinal glutathione and exacerbation of neuronal dysfunction [47].

Overall, in a translational perspective the present findings suggest that preventive treatments based on NACET may be effective in avoiding retinal complications in diabetic subjects. Whether NACET may be also effective in a curative setting, that is administered in patients with overt DR, cannot be inferred by the present results and the question needs to be explored in further experiments in Akita mice treated with NACET after the onset of early signs of DR.

In conclusion, our data show that NACET is an effective antioxidant not only as a cell-permeable GSH precursor but also as a trigger of the KEAP1-NRF2 oxidative defence pathway, and that it is a promising drug for the prevention of AMD and DR.

## 4 Methods

### 4.1 Chemicals

NAC (C5H9NO3S, MW 163,19 g/mol; #A7250; Sigma-Aldrich; Merck KGaA, Darmstadt, Germany) and NACET (C7H13NO3S, MW 191.2 g/mol; #O32426, Lot#:AC99952A; Advanced ChemBloks, Hayward, CA, USA) were prepared from powder to a final 200 mM concentration in Milli-Q water or ethanol. The corresponding vehicle was used for negative control samples. For NACET chemical characterization see Giustarini et al., 2012 [23]. We tested NACET from many different suppliers and used the NACET with the best percentage of reduced NACET versus NAC or oxidized NACET (NACETSS). Specifically, we measured 95.9 % NACET, 1.3 % NAC, 2.8 % NACETSS and 0.0 % NACSS for the lot used. BSO (l-Buthionine-sulfoximine; #B2515; Sigma-Aldrich; Merck KGaA, Darmstadt, Germany) were prepared to a final 100 μM concentration in Milli-Q water, then 0.22 μm filtered. Hydrogen peroxide (#95299; Fluka Chemie, Buch, Switzerland) was diluted in Milli-Q water just prior to use.

### 4.2 Animals and treatment with NACET

Young (8–20 weeks old) and old (80–110 weeks old) male wild-type C57BL6/J mice were group housed and maintained in a Specific Opportunist Pathogen Free (SOPF) animal facility in Fritz Lipmann Institute with 12 h of light/dark cycle and fed with a standard mouse chow at Temperature 20 ± 2 °C, rlH 55 % ± 15. NACET was supplemented with drinking water. To achieve an oral dosage of 50 mg/kg body weight/day of NACET, a solution 0.25 mg/mL NACET has been used, which has been prepared fresh every two day and administered to mice in place of drinking water, to use in the placebo group. This concentration is based on the average water consumption per mouse (5 mL/day measured in our animal facility). After 5 days mice have been sacrificed by CO2 euthanasia, then we proceed with the dissection of the eyes to collect the retinas as described in Simmons and Fuerst, 2018 [48]. One of the retinas of each mouse were homogenized in 300 μL of a 4 % (w/v) solution of ice-cold trichloroacetic acid (TCA; Sigma-Aldrich; Merck KGaA, Darmstadt, Germany) containing 1 mM K3EDTA and homogenized in ice by means of a tissue grinder. Samples were stored at −80° until analyzed to measure the concentration of Cys and GSH, through High Performance Liquid Chromatography (HPLC) analysis as previously described [17]. The GSH concentration was normalized for protein content. The second retina of each mouse was used for RNA-seq analysis. Experiments were conducted according to protocols approved by the state government of Thuringia Thüringer Landesamt für Verbraucherschutz (TLV) authority (license numbers: TG/J-0002858/A; TG/J-0003616/A; TG/J-0003681/A; FLI-20-005).

Akita (C57BL/6-*Ins2*^*Akita*^/J) is a monogenic mouse model carrying a spontaneous mutation in the insulin 2 gene [21]. *Ins2*^*Akita*^ mutation would lead to incorrect folding of the insulin protein thus producing toxicity in pancreatic β cells, reduced β cell mass and reduced insulin secretion typical of type 1 diabetes. Akita mice were used to test the effect of NACET administration as compared with that obtained in C57BL/6J mice (WT), used as normoglycemic control. Only male mice were involved in the study as female Akita mice are reported to develop a milder and less consistent diabetic phenotype [49]. All the procedures were performed in compliance with the ARVO Statement for the Use of Animals in Ophthalmic and Vision Research, the EU Directive (2010/63/EU), and the Italian guidelines for animal care (DL 26/14; Italian Ministry of Health, decree number 606/2024-PR). Twenty-two WT and 22 Akita mice were obtained from The Jackson Laboratories (Bar Harbor, ME, USA). At 4 weeks of age, WT and Akita mice were randomly assigned to either vehicle- or NACET-treatment group (11 mice per group). NACET was dissolved in drinking water at the concentration of 0.33 mg/mL to obtain a daily oral intake of 50 mg/kg. Mice received the oral administration of vehicle (water) or NACET everyday up to the 24th week of age. During the treatment period, mice underwent a longitudinal assessment of blood glucose level using a OneTouch Ultra glucometer (LifeScan Inc., Milpitas, CA, USA). A longitudinal assessment of retinal activity and retinal/vascular morphology was performed as well (see below). At the 24th week of age, mice were euthanized by cervical dislocation and retinas were harvested for further analyses.

### 4.3 Longitudinal electrophysiological assessment of retinal activity

Retinal activity was assessed by performing electroretinogram (ERG) recordings at 8, 12, 16 and 24 weeks of age. At each time-point, WT or Akita mice either untreated or treated with NACET were subjected to a recording routine providing the analysis of the overall retinal activity with scotopic ERG (scERG) and the analysis of retinal ganglion cell (RGC) activity with pattern ERG (PERG). After overnight dark-adaption, mice were anesthetized with an intraperitoneal injection of Avertin (1.2 % w/v Avertin, 0.02 mL/g body weight; Sigma‐Aldrich, St. Louis, MO, USA) and gently restrained in a custom-made stereotaxic apparatus allowing an unobstructed visual field. Body temperature was maintained at 37 °C by means of a feedback-controlled heating pad and corneal moisture was ensured by adding balanced saline solution before and during the recording session. Silver-silver chloride electrodes made of 0.2 mm loop-shaped wires were carefully laid on the corneal surface of each eye using micro manipulators. A stainless-steel needle for each eye was inserted in the scalp and used as references electrode. A further stainless-steel needle was used as a ground electrode after being inserted at the tail root.

In each recording session, scERG was first performed using a commercially available ERG setup (Retimax Advanced, CSO, Firenze, Italy) and a 10 cd s/m^2^ flash stimulus delivered with a Ganzfeld bowl. Five consecutive signals recorded simultaneously from each eye were averaged to reduce noise after amplification (5000-fold) and band-pass filtered (1–100 Hz). In the deriving waveform, the amplitude of the a-wave (baseline to trough) and that of the b-wave (trough to peak) were measured.

The scERG session was followed by PERG recordings. Using the same electrode setup, a visual stimulus consisting of black and white (98 % contrast) bars contrast-reversing at 1 Hz temporal frequency and 0.05 cyc/deg spatial frequency was administered with a 19″ light emitting diode display (area: 74° × 62°) aligned to the mouse cornea and spaced 25 cm from the mouse eye. Each pattern reversal-deriving signal was amplified (10 000-fold) and band-pass filtered (1–30 Hz). Overall, the responses deriving from 900 consecutive pattern reversals were averaged to reduce noise contamination by a factor of √900 = 30. PERG responses were analyzed by detecting the early positive peak (P50), and the belated negative trough (N95) in each PERG waveform to then retrieve the P50–N95 amplitude (hereinafter referred as PERG amplitude) as a sensitive measure of RGC activity ensuring an optimized dynamic range of measures and signal-to-noise ratio. Implicit time of the PERG response was also longitudinally analyzed as time-to-P50 (hereinafter referred as PERG latency).

### 4.4 Longitudinal analysis of retinal layer thickness

Retinal layer thickness was analyzed *in vivo* at each time point by performing image-guided optical coherence tomography (OCT) using the Micron IV system (Phoenix Research Laboratories, Pleasanton, CA, USA). Mice were anesthetized by intraperitoneal injection of avertin and laid on a custom-made holder allowing the relative orientation of the eye with the instrument probe. Mydriasis was induced by 1 % tropicamide and 2 % hydroxypropylmethylcellulose drops were used to avoid eye drying. After the optic nerve head was aligned with the probe axis, OCT images were acquired by image-guided circular scans (550 μm diameter) around the optic nerve head. The segmentation of retinal layers and the quantification of layer thickness was performed using Insight software (Phoenix Research Laboratories). The whole retina thickness was calculated from the retinal nerve fiber layer (RNFL) to the photoreceptor outer segment layer. The retina was further segmented to calculate the thickness of inner retinal layers (IRL), including RNFL, GCL, inner plexiform layer (IPL) and inner nuclear layer (INL), and the thickness of outer retinal layers (ORL), including outer plexiform layer (OPL), outer nuclear layer (ONL), photoreceptor inner and outer segments. Both eyes were examined, and results were averaged.

### 4.5 Whole-mount retina immunofluorescence and quantitative analysis

Briefly, isolated retinas were immersion-fixed in 4 % w/v paraformaldehyde and stored at 4 °C in 25 % sucrose in 0.1 M PBS. Retinas underwent whole-mount immunostaining for RNA-binding protein with multiple splicing (RBPMS), using a rabbit polyclonal antibody (NBP2-20112; Novus Biologicals, Bio-Techne srl, Milano Italy) at 1:100 dilution for 72 h. Retinas were then incubated for 48-h with Alexa Flour 488 anti-Rabbit IgG (H + L) (#A21206; Thermo Fisher Scientific, Waltham, MA, USA) at 1:200 dilution. After processing, retinas were analyzed with an epifluorescence microscope (Ni-E; Nikon Europe, Amsterdam, TheNetherlands) equipped with a 20× plan achromat objective, a digital camera (DS-Fi1c; Nikon Europe), and a motorized stage for organ whole-mount reconstruction. The derived images were automatically analyzed for the RBPMS-positive cell density following the sampling of four radially opposite images at two different radial eccentricities (center = 0.5 mm, periphery = 4 mm from the optic disc) in order to determine the average density of RGCs in peripheral and central retina.

### 4.6 Cell culture

ARPE-19 cell line [50] was grown in DMEM/F12 (1:1) containing 10 % (v/v) fetal bovine serum (FBS) and penicillin and streptomycin (all reagents were purchased from Euroclone, Milan, Italy). Cells were free of mycoplasma, confirmed by the MycoAlert mycoplasma detection kit (Lonza, Walkersville, MD, USA). The ARPE-19, a spontaneously arising retinal pigment epithelia (RPE) cell line, was used for these experiments. ARPE-19 is widely used to study retinal cell biology, pathological conditions, and pharmacology.

### 4.7 Crispr/Cas9 editing of NFE2L2

To obtain that, ARPE-19 were transduced with lentiviral particles for the expression of the endonuclease Cas9 and a guide RNA (gRNA) specific for the exon 5 of NFE2L2 (NM_006164.5). As negative control, other cells were transduced with particles missing the gRNA sequence in the viral genome. The plasmid LentiCRISPR v2 (pLCv2) (Addgene, plasmid #52961) was used to obtain NEF2L2 editing in the ARPE-19 cell line. It contains two expression cassettes for the hSpCas9 and the chimeric guide RNA. The vector was digested using *Bsm*BI (NEB, Ipswich, MA, USA) and a pair of annealed synthetic oligonucleotides was cloned into the single guide RNA scaffold. The oligonucleotides were designed based on a target site sequence of 23 pb. The online tool named CHOPCHOP (https://chopchop.cbu.uib.no/) was used to design the sgRNA. The sequences of the oligonucleotides used are:

sgRNA forward: 5′ CACCGCATTAATTCGGGATATACGTAGG 3’

sgRNA reverse: 5′ AAACCCTACGTATATCCCGAATTAATGC 3’

The underlined sequences form the two overhangs necessary to clone in the correct orientation the double stranded oligonucleotides in the pLCv2 vector digested by *Bsm*BI. The proper sgRNA insertion was checked by Sanger sequencing.

### 4.8 Plasmids and site-directed mutagenesis

For the RNA interference-mediated knockdown of KEAP1, we used the lentiviral plasmid pLKO.1 from the TRC shRNA library (Sigma-Aldrich; Merck KGaA, Darmstadt, Germany) expressing specific shRNA for 3’ UTR of human KEAP1 (#TRCN0000155340). Plasmid for KEAP1 (NM_012289) expression in KEAP1-silenced cells was purchased from ORIGENE (#SC111946; Rockville, MD, USA). The point mutations in human KEAP1 cDNA were introduced using the QuickChange XL Site-Directed Mutagenesis kit (Agilent, Santa Clara, CA, USA). For mass spectrometry analysis, the plasmid Flag-Keap1 (#28023; Addgene, Watertown, MA, USA) was transfected for the expression of C-terminal 3xFlag KEAP1 [51].

### 4.9 Cell transfection

For mass spectrometry analysis, 2.5x106 HEK-293 LentiX cells were plated in four 100-mm culture dishes and transfected 24 h later with 12 μg/plate of Flag-Keap1 plasmid and 48 μL/plate of Transport 5 Transfection Reagent (#26008; Polysciences Inc., Warrington, PA, USA) according to the manufacturer's instructions. After 48 h, the 2 cell dishes were treated with 1 mM NACET for 2 h and then all 4 dishes were lysed in 1 mL/plate of RIPA buffer (50 mM Tris-HCl pH 7.2, 100 mM NaCl, 1 % (v/v) Triton X100, 1 % (w/v) deoxycholic acid, 0.1 % (w/v) SDS, 50 NaF, 2 mM EDTA) with proteinase inhibitor cocktail (Sigma-Aldrich; Merck KGaA, Darmstadt, Germany) by gentle shearing with an insulin syringe and needle.

For transfection described in Fig. 3, 4x10 [5] control (ctrl.) or edited (NRF2 ΔC) ARPE-19 cells were plated in 35-mm culture dishes and transfected with 0.3 μg of pRL-SV40 vector, and 3 μg of pGL4.37 (lucP/ARE/Hygro) vector, and 12 μL of Transport 5 Transfection Reagent following the manufacturer's specifications. The pGL4.37 (lucP/ARE/Hygro) vector (Promega Corp., Madison, WI, USA) contains four copies of an ARE response element that drives transcription of the luciferase gene luc2P and was used to evaluate the NRF2 activity. The pRL-SV40 vector (Promega Corp., Madison, WI, USA) contains the Renilla luciferase gene under the control of a constitutive promoter and was used as internal control of transfection efficiency. 24 h after transfection the medium was changed, and cells were treated with NACET. After 24 h, cells were washed twice with PBS and lysed in 1x PLB, then, by means of a scraper, cells were mechanically removed from the plate and collected in a 1.5 mL centrifuge tube. Finally, the samples were sheared using Diagenode's Bioruptor 300 at HIGH setting, 5 cycles of 30 s and centrifuged at 16200×*g*, for 5 min at 4 °C, the supernatant was recovered and stored at −20 °C until analyzed.

For transfection described in Fig. 4, ARPE-19 cells silenced for KEAP1 by lentiviral transduction were transfected with 0.06 μg of pRL-SV40 vector, 0.54 μg of pGL4.37 (lucP/ARE/Hygro) vector, 0.6 μg of pCMV6-KEAP1 vector for the expression of KEAP1 wt or mutants, and 8 μL of Transport 5 Transfection Reagent. After 8 h the medium was changed with growth medium and the cells were treated or not with 1 mM NACET. Cells were lysed 24 h post treatment.

### 4.10 Dual-luciferase assay

After cell lysis, luminescence was measured by means of Dual Luciferase Reporter (DLR) Assay System kit (Promega Corp., Madison, WI) and the luminometer Turner Designs (model TD-20/20; Sunnyvale, CA, USA). All reagents were prepared and used according with manufacturer's specifications. The data are represented as the ratio of Firefly to Renilla luciferase activity (Fluc/Rluc).

### 4.11 KEAP1 mass spectrometry analysis

For mass spectrometry analysis, 2 mL of lysates of NACET-treated and untreated HEK-293 LentiX cells transfected with the plasmid Flag-Keap1 was affinity purified with 400 μL of Anti-FLAG M2 Magnetic Beads (#M8823; Sigma-Aldrich; Merck KGaA, Darmstadt, Germany) following the manufacturer's specifications. Flag-KEAP1 was eluted twice with 1 mL of 0.1 M Glycine HCl, pH 3. After the elution, 200 μL of 0.5 M Tris HCl pH 7.4/1.5 M NaCl, was added to re-equilibrate to neutral pH. Mass spectrometry analysis was kindly performed by Dr. Laura Salvini (Toscana Life Sciences, Siena). Protein samples were desalted and digested using trypsin (Promega). The resulting peptides were then analyzed using an Ultimate 3000 RSLC nano coupled to a Q-Exactive HF-X Orbitrap mass spectrometer (Thermo Fisher Scientific) equipped with an Easy Spray ESI ion source. LC-MS/MS analyses were performed using Q-Exactive HF-X Orbitrap mass spectrometer (Thermo Scientific). The peptide separation was carried out at 35 °C using a PepMap RSLC C18 column, 75 μm × 15 cm, 2 μm, 100 Å (Thermo Fisher) at a flow rate of 300 nL/min. The mobile phases A and B used for the analysis were 0.1 % formic acid in water and 0.1 % formic acid in acetonitrile, respectively. The gradient started with 5 % of B and then it was increased up to 90 % in 120 min. The experiment was performed using a data dependent analysis (DDA) setting to select the “top twenty” most-abundant ions for MS/MS analysis. The MS/MS spectra were obtained in “data-dependent scan” mode. Precursor ions were selected within an isolation window of 1.4 *m*/*z*. Fragment ion spectra were detected in the orbitrap at a resolving power of 30 000. MS/MS was performed with a dynamic exclusion of 20 s and a total cycle time of 3 s. LC-MS/MS data were analyzed with BioPharmaFinder software (version 2.0, ThermoFisher Scientific). For the elaboration cysteinylation, glutathionylation, and conjugation with N-acetylcysteine on cysteine were considered as variable modifications on the protein sequence. Only peptides bearing modifications identified by the MS/MS spectra, a confidence score greater than 95 % and mass tolerance within ±2 ppm were considered.

### 4.12 Lentiviral packaging and transduction

Lentiviral particles were produced by transfection of HEK-293 LentiX cells as previously described [52]. For transduction, ARPE-19 cells were plated in 6-well plates at a concentration of 1 × 10^5^ cells/well. After 24 h, the medium was replaced with complete medium and 0.025–0.250 mL Lenti-X 293 T supernatant containing lentiviral particles and 4 μg/mL polybrene (hexadimethrine bromide; #H9268; Sigma-Aldrich; Merck KGaA, Darmstadt, Germany) per well. After 24 h, the medium was changed, and after another 48 h, the cell extracts were analyzed by Western blot or cultured for transient transfection and dual-luciferase assay. For gene editing, selection with puromycin (2 μg/mL) was performed from 48 h after transduction. Subsequently, clonal selection was performed on the selected cells. The clones were analyzed by Western blot and the clone with the designation C1 was selected. Exon 5 of NFE2L2 was amplified by PCR using Q5 high-fidelity DNA polymerase (NEB, Ipswich, MA, USA). The amplified amplicon was cloned into the TOPO vector using the Zero Blunt™ TOPO™ PCR Cloning Kit (Thermo Fisher Scientific, Waltham, MA, USA) and the sequences of the alleles were verified by Sanger sequencing of 10 plasmid clones obtained.

### 4.13 Immunofluorescence

1.5 × 10^4^ cells/well cells were plated in 8 well chamber slides (#154534, Nunc Lab-Tek II Chamber Slides; Thermo Fisher Scientific, Waltham, MA, USA). After 24 h cells were treated with 1 mM NACET for the indicated time points before fixation. Cells were permeabilized in PBS/0.1 % (v/v) Triton X-100 (PBST), saturated using PBST/1 % BSA/5 % (v/v) donkey serum for 30 min and incubated with the diluted (1:500) anti-NRF2 (#EP1808Y; Abcam, Cambridge, UK) and anti-α-Tubulin (#T5168; Sigma-Aldrich; Merck KGaA, Darmstadt, Germany) primary antibodies in PBST-1 % BSA. Alexa Flour 488 anti-Rabbit IgG (H + L) (#A21206; Thermo Fisher Scientific, Waltham, MA, USA) and Alexa Flour 568 Anti-Mouse IgG (H + L) (#A10037; Thermo Fisher Scientific, Waltham, MA, USA) were used as secondary antibodies. 0.1 μg/mL DAPI was used to stain the nuclei. The images were taken using a ZEISS Apotome 3 (ZEISS, Baden-Württemberg, Germany) and analyzed with ImageJ software, to obtain the mean fluorescence intensity of cells expressing a nuclear protein of interest [53].

### 4.14 RNA extraction and isolation

Total RNA was extracted and isolated from ARPE-19 or mouse retinas using EuroGOLD TriFast™ (EuroClone, Milan, Italy) according to manufacturer's specifications.

### 4.15 RT-qPCR, mRNA sequencing library preparation and data analysis

For RT-qPCR, total RNA was extracted using RNeasy Plus Mini Kit (Qiagen, Hilden, Germany), according to the manufacturer's instruction and gene expression was evaluated by means of QuantiNova SYBR Green RT-PCR Kit and the Rotor-Gene Q thermocycler (Qiagen, Hilden, Germany). Primers for RT-qPCR are reported in Supplementary Table 2. For gene expression profiling, total RNA was purified using EuroGOLD TriFast™ (Euroclone, Milan, Italy). RNA quantity and quality were evaluated by Nanodrop 8000 (Thermo Fisher Scientific, Waltham, MA, USA) and Fragment Analyzer (Advanced Analytical Technologies, Heidelberg, Germany) using the DNF-471 Standard Sensitivity RNA Analysis Kit (15 nt) (Agilent Technologies, Santa Clara, CA, USA). RNA samples were further processed for mRNA-seq library preparation using the TruSeq RNA Sample preparation v2 kit according to the manufacturer's instructions (Illumina, San Diego, CA, USA). The sequencing was done using an Illumina NextSeq 500 sequencer (single end). Fastq files quality was checked using FastQC v0.12.1. The fastq files were mapped to the mm9 or hg19 genome using TopHat v2.1.0 [54] with the following parameters --bowtie1 --no-coverage-search -a 5. The number of reads covered by each gene was calculated using HTSeq-Count 0.11.2 [55] with -s no -a 0 -t exon -m intersection-nonempty parameters. All of the rRNA genes were removed from the count data before the downstream analysis. For calculating differentially expressed genes (DEGs) and normalized count, DESeq2 R package (v1.20.0) [56] was used with the default parameters. For the PCA plot, the normalized count was used, and the genes were filtered with the following criteria, count>10 in at least 5 samples, IQR (interquartile range) of the log2 transform count ≥ 1.5. To calculate the PCA, the log 10 transformed count was used in prcomp R function with center = TRUE, scale. = TRUE parameters. PC1 and PC2 were used for the plotting using fviz function (factoextra_1.0.7 R package). Ingenuity Pathway Analysis (IPA v45868156, Qiagen), was used for functional and pathway as well as upstream regulators analysis [57] with DEGs (padj<0.05). For GO analysis, DEGs gene were mapped to identifier GO term using annFUN.org function (topGO v2.50.0) with whichOnto = “BP”, feasibleGenes = NULL, mapping = “org.Hs.eg.db” for human and “org.Mm.eg.db” for mouse. Subsequently, topGOdata object was created using new (method package from R base) function with classs = “topGOdata”, ontology = “BP”, annot = annFUN.GO2genes, nodeSize = 10 parameters. For KEGG analysis, the gene Enterz ID for DEGs were extracted using mapIds function (AnnotationDbi_1.68.0 R package). The enriched KEGG terms were calculated using enrichKEGG function (clusterProfiler_4.14.3 R package) with the following parameters, keyType = “ncbi-geneid”, pAdjustMethod = “fdr”, minGSSize = 5. Heatmaps were plotted using pheatmap (pheatmap_1.0.12 R package) or heatmap.2 function (gplots_3.2.0 R package).

### 4.16 Western blot analysis

Protein extracts from either cultured cells or retinal tissue were prepared as previously described [53,58]. Primary and secondary antibodies used for immunoblotting are reported in Supplementary Table 3. Chemiluminescent bands were detected by means of ImageQuant™ LAS 4000 (GE Healthcare Life Science, Chicago, IL, USA) or ChemiDoc XRS+ (Bio-Rad).

### 4.17 Analysis of intracellular low molecular mass thiols (LMM-SH)

To measure the intracellular levels of NAC, Cys, γ-GluCys, CysGly and GSH from *in vitro* experiments with ARPE-19 cells, the culture medium was removed, and the cells were washed twice with PBS at 4 °C, lysed with 0.5 mL of a 4 % (w/v) solution of ice-cold TCA containing 1 mM K3EDTA and collected after scraping. For the ex vivo experiments, we homogenized the retinas in 9 μL of 4 % (w/v) solution of ice-cold TCA containing 1 mM K3EDTA per each 1 mg of tissue. Samples were either immediately analyzed or stored at −80 °C until analyzed. Measurements were always carried out within 5 days from sample preparation. After labelling of the –SH group with the fluorescent probe monobromobimane (mBrB) (Calbiochem, La Jolla, CA, USA) as previously described [17]. An Agilent series 1100 HPLC (Agilent Technologies, Milan, Italy) equipped with diode array and a fluorescence detector was used for all determinations. For the normalization, protein content was measured by the Bradford assay after protein pellet resuspension in 0.1 N NaOH. Bovine serum albumin was used as standard.

### 4.18 Statistical analysis

The data analysis was performed using Prism 9 statistical software (GraphPad Software Inc., San Diego, CA, USA). Evaluation of the data was conducted one-way or two-way ANOVA, Kruskal-Wallis tests. ∗p < 0.05, ∗∗p < 0.01, ∗∗∗p < 0.001, ∗∗∗∗p < 0.0001.

## CRediT authorship contribution statement

**Giulia Realini:** Formal analysis, Investigation, Methodology, Validation, Writing – review & editing. **Rosario Amato:** Formal analysis, Investigation, Methodology, Validation, Writing – review & editing. **Mahdi Rasa:** Data curation, Formal analysis, Writing – review & editing. **Roberto Ceccatelli:** Investigation, Writing – review & editing. **Angela Cannavale:** Investigation, Writing – review & editing. **Laura Bottoni:** Investigation, Writing – review & editing. **Federico Marchini:** Investigation, Writing – review & editing. **Alberto Minetti:** Investigation, Writing – review & editing. **Daniela Giustarini:** Investigation, Writing – review & editing. **Alessio Canovai:** Investigation, Writing – review & editing. **Alberto Melecchi:** Investigation, Writing – review & editing. **Ines Elia:** Investigation, Methodology, Writing – review & editing. **Anna Krepelova:** Investigation, Writing – review & editing. **Francesco Annunziata:** Investigation, Writing – review & editing. **Maurizio Cammalleri:** Investigation, Writing – review & editing. **Ranieri Rossi:** Methodology, Resources, Writing – review & editing. **Gian Marco Tosi:** Conceptualization, Writing – review & editing. **Maurizio Orlandini:** Investigation, Writing – review & editing. **Mario Chiariello:** Funding acquisition, Resources, Writing – review & editing. **Francesco Neri:** Formal analysis, Methodology, Writing – review & editing. **Massimo Dal Monte:** Conceptualization, Funding acquisition, Methodology, Resources, Supervision, Writing – review & editing. **Federico Galvagni:** Conceptualization, Formal analysis, Funding acquisition, Investigation, Methodology, Project administration, Resources, Supervision, Validation, Visualization, Writing – original draft, Writing – review & editing.

## Declaration of competing interest

The authors declare that they have no known competing financial interests or personal relationships that could have appeared to influence the work reported in this paper.

## Data Availability

Raw and processed RNA-seq data are available in the Gene Expression Omnibus under accession numbers GSE299875, GSE299876, GSE299877, GSE299879. Source data files for the analyses are available in the Supplementary Information files. All other data are available from the corresponding author upon reasonable request. Source data are provided with this paper.
