# Supplementary material for: N-acetyl-l-cysteine ethyl ester (NACET) induces the transcription factor NRF2 and prevents retinal aging and diabetic retinopathy
Source: Redox Biol. 2025 Nov 3;88:103914. doi: 10.1016/j.redox.2025.103914 (PMC12793733; doi:10.1016/j.redox.2025.103914)
Supplement: Multimedia component 10 [file mmc10.pdf]

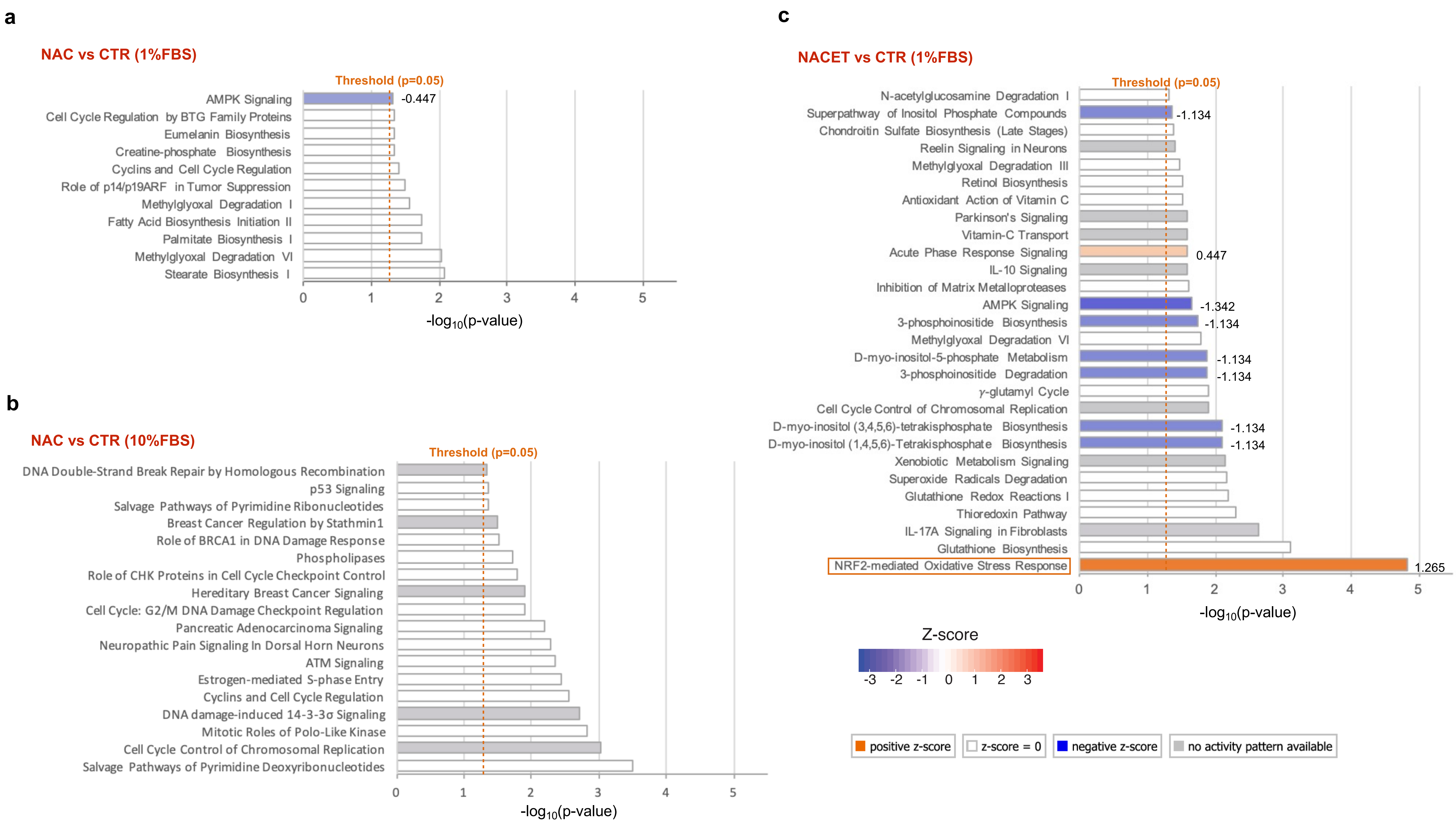

Supplementary Fig. 1. IPA of DEGs for NAC (a and b) and NACET (c) -treated vs. control cells under 1% FBS (a, c) and 10% FBS (b) conditions. The values of the positive and negative z-scores are indicated. The NRF2 signaling pathway in NACET-treated cells is the most significantly regulated pathway in both serum conditions.

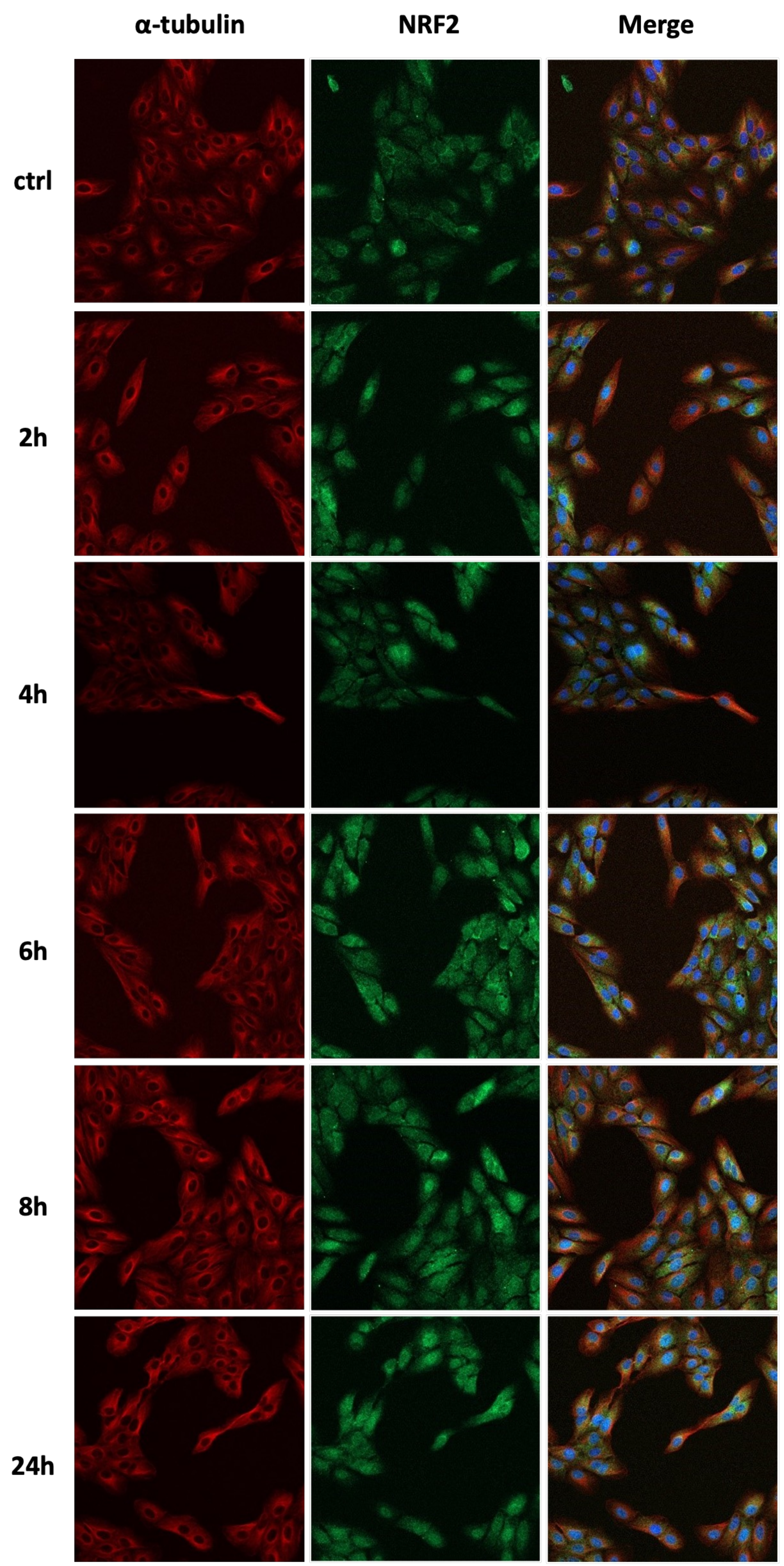

Supplementary Fig. 2. *NRF2 nuclear localization in ARPE-19 cells after NACET treatment.* Immunofluorescence staining of α-tubulin, NRF2 and merge images at 0 (ctrl), 2, 4, 6, 8 and 24 hours after NACET 1 mM treatment.

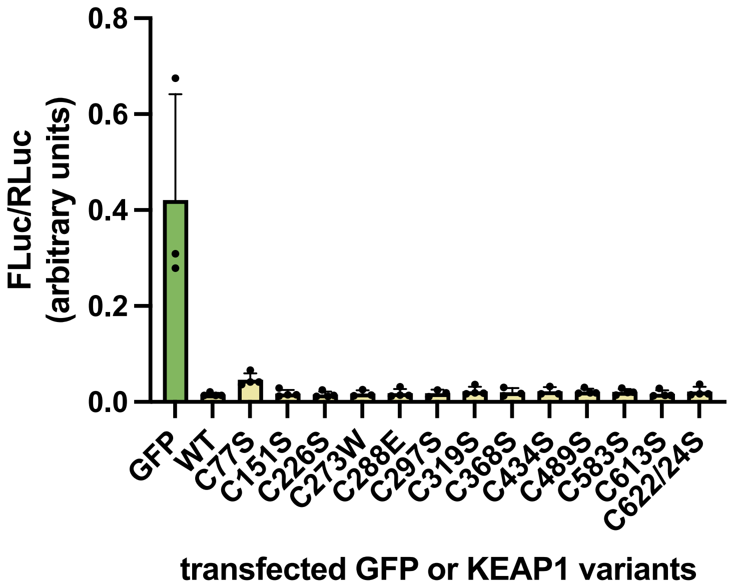

Supplementary Fig. 3. KEAP1-silenced ARPE-19 cells were transfected with ARE-Firefly Luciferase and RSV-Renilla Luciferase plasmids, along with a plasmid expressing shRNA-resistant KEAP1 (wild-type or mutant, as indicated) or GFP as a negative control.

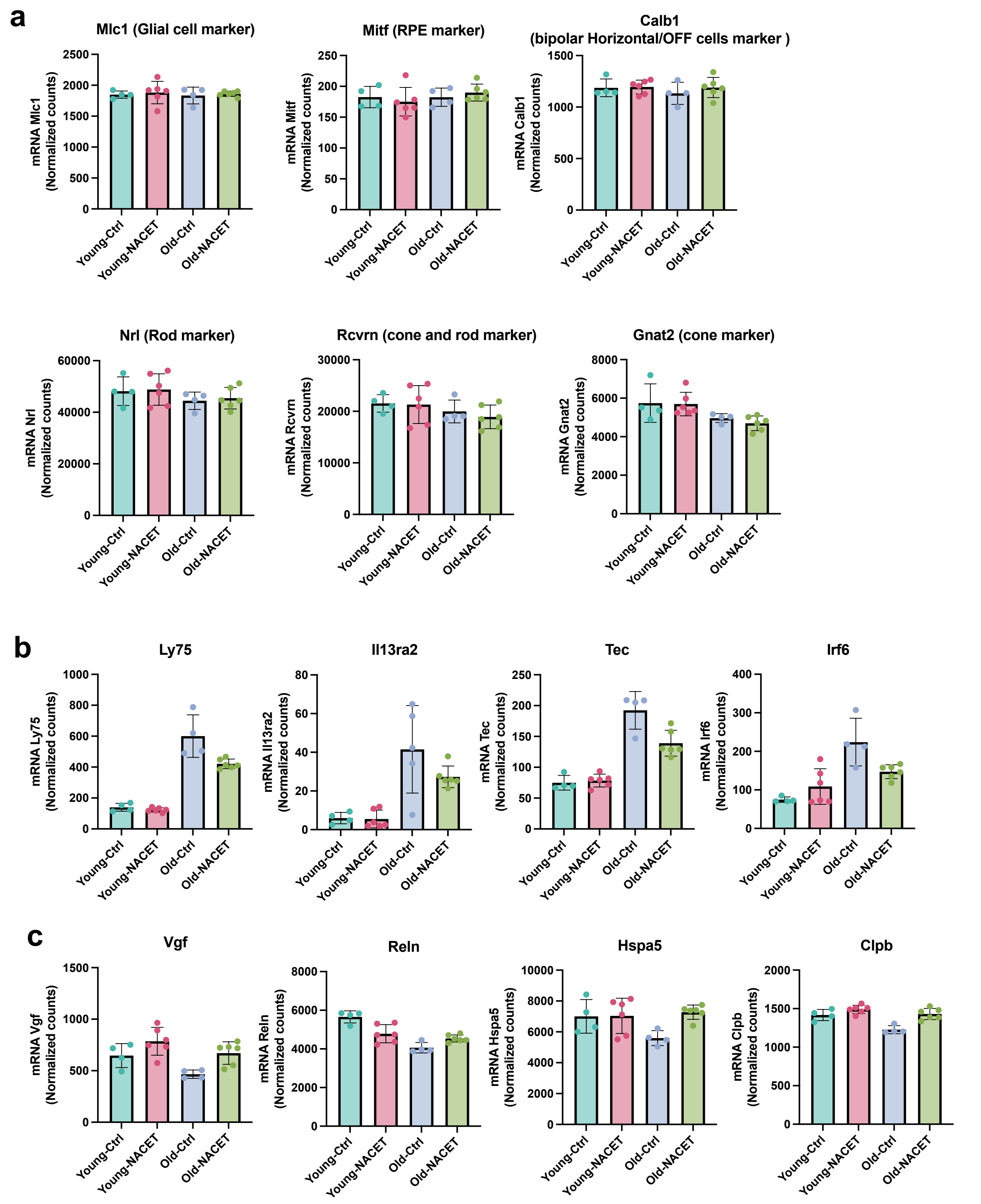

Supplementary Fig. 4. *DESeq2 mean normalized counts from transcriptome analysis of young and aged retinas of NACET-treated and control animals.* **a**, Marker genes of retinal cells. **b**, DEGs of aged retina rescued by NACET treatment involved in inflammation. **c**, DEGs of aged retina rescued by NACET treatment involved in UPR and/or downregulated in aging and/or neurodegenerative diseases.

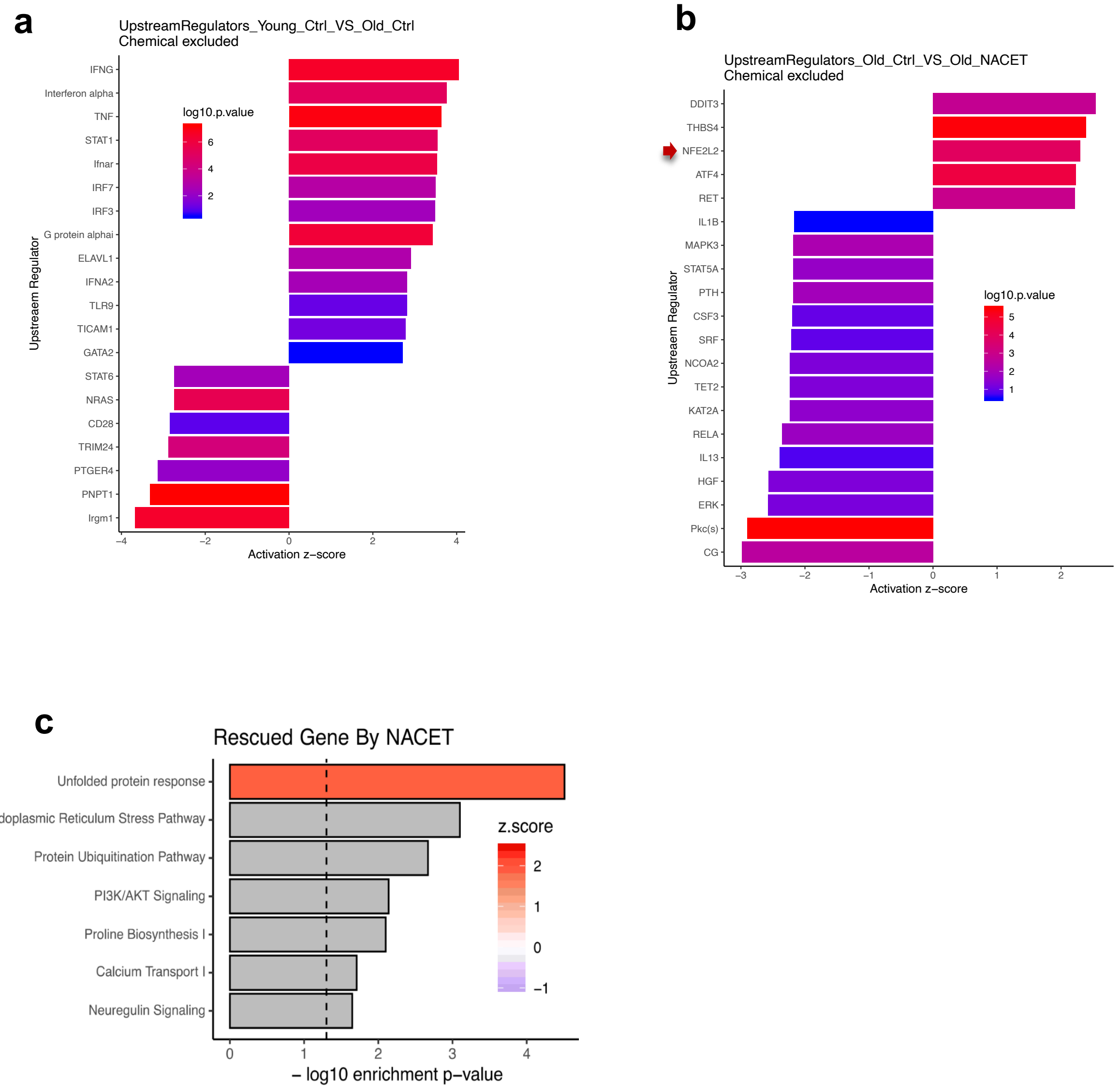

Supplementary Fig. 5. *IPA Upstream Regulator Analyses of DEGs between* **a**, retinas of young and old mice and **b**, retinas of untreated and NACET-treated old mice. **c**, IPA analysis of the 57 DEGs in aging that were rescued by NACET.

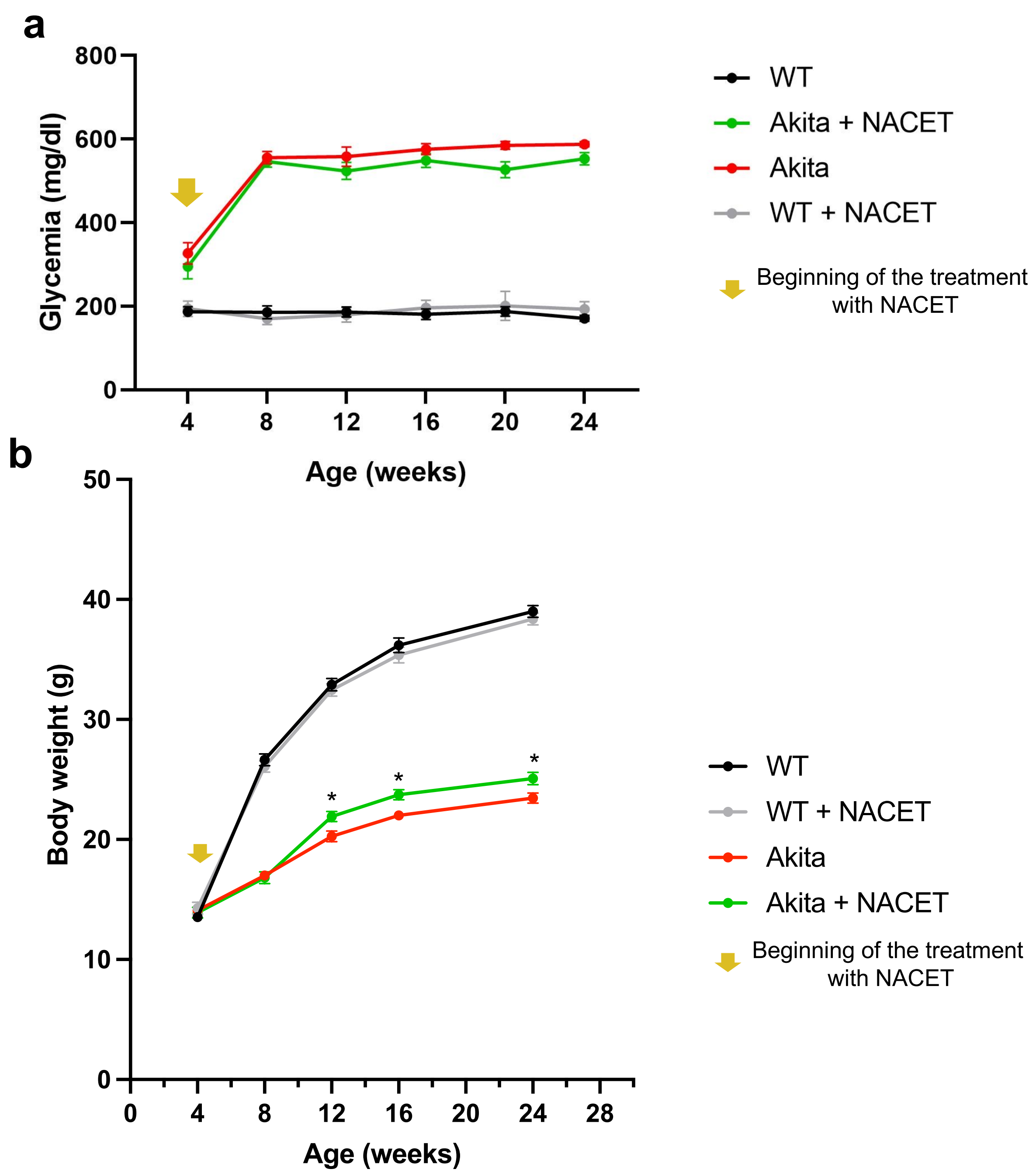

Supplementary Fig. 6. *Non-fasting glycemia (**a**) and body weight (**b**) measurements for wt and Akita mice treated and untreated with NACET.* Measurements are the means ± SD. Differences between Akita and NACET-treated Akita groups were tested using two-way ANOVA followed by Šídák's multiple comparisons test. * $p < 0.05$.

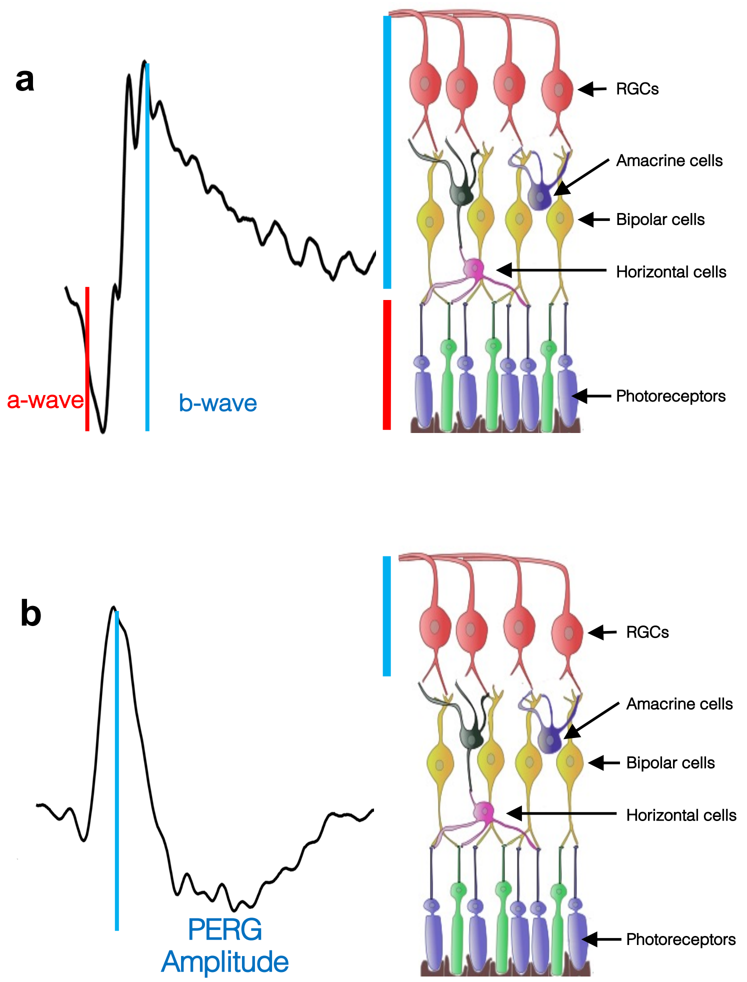

Supplementary Fig. 7. *ERG and OCT analysis of the retina.* **a**, Representation of an ERG showing an initial wave of hyperpolarisation (a-wave) followed by a wave of depolarisation (b-wave) in response to high light intensity. The a-wave results from photoreceptor hyperpolarisation and records photoreceptor activity. The b-wave results from the depolarisation of most inner retinal neurons. **b**, Representation of an ERG showing the recording of the PERG wave amplitude, which measures the RGC activity.
