# Supplementary material for: N-acetyl-l-cysteine ethyl ester (NACET) induces the transcription factor NRF2 and prevents retinal aging and diabetic retinopathy
Source: Redox Biol. 2025 Nov 3;88:103914. doi: 10.1016/j.redox.2025.103914 (PMC12793733; doi:10.1016/j.redox.2025.103914)
Supplement: Multimedia component 18 [file mmc18.docx]

| **Symbol** | **Gene ID** | **Full name** | **AGING** | **NACET** |
| --- | --- | --- | --- | --- |
| A930031H19Rik | 77835 | RIKEN cDNA A930031H19 gene | -1,115 | 0,980 |
| Spc25 | 66442 | SPC25, NDC80 kinetochore complex component, homolog | -0,645 | 0,316 |
| Htra1 | 56213 | HtrA serine peptidase 1 | -0,528 | 0,355 |
| Reln | 19699 | Reelin | -0,479 | 0,163 |
| Vgf | 381677 | VGF nerve growth factor inducible | -0,475 | 0,527 |
| D630045J12Rik | 330286 | RIKEN cDNA D630045J12 gene | -0,434 | 0,209 |
| Aldh18a1 | 56454 | Aldehyde dehydrogenase 18 family, member A1 | -0,390 | 0,465 |
| Mir670hg | 414123 | MIR670 host gene (non-protein coding) | -0,388 | 0,384 |
| Slc1a4 | 55963 | Solute carrier family 1, member 4 | -0,376 | 0,288 |
| Lrfn2 | 70530 | Leucine Rich Repeat And Fibronectin Type III Domain Containing 2 | -0,367 | 0,359 |
| Mrtfa | 223701 | Myocardin related transcription factor A | -0,333 | 0,239 |
| Hspa5 | 14828 | Heat shock protein 5 | -0,325 | 0,380 |
| Calr | 12317 | Calreticulin | -0,284 | 0,368 |
| Tuba4a | 22145 | Tubulin, alpha 4A | -0,275 | 0,246 |
| Fbxw8 | 231672 | F-box and WD-40 domain protein 8 | -0,262 | 0,197 |
| Hspa8 | 15481 | Heat shock protein 8 | -0,246 | 0,430 |
| Ankrd13b | 268445 | Ankyrin repeat domain 13b | -0,243 | 0,200 |
| Mib2 | 76580 | Mindbomb E3 ubiquitin protein ligase 2 | -0,237 | 0,228 |
| Scg2 | 20254 | Secretogranin II | -0,232 | 0,235 |
| Mars | 216443 | Methionine-tRNA synthetase 1 | -0,225 | 0,224 |
| Hsp90ab1 | 15516 | Heat shock protein 90 alpha (cytosolic), class B member 1 | -0,222 | 0,224 |
| Ap3b2 | 11775 | Adaptor-related protein complex 3, beta 2 subunit | -0,220 | 0,127 |
| Bag6 | 224727 | BCL2-associated athanogene 6 | -0,219 | 0,133 |
| Ap1m1 | 11767 | Adaptor-related protein complex AP-1, mu subunit 1 | -0,218 | 0,221 |
| Clpb | 20480 | ClpB caseinolytic peptidase B | -0,207 | 0,222 |
| Ank2 | 109676 | Ankyrin-2 | -0,185 | 0,107 |
| Drosha | 14000 | Drosha, ribonuclease type III | -0,181 | 0,142 |
| Atp2a2 | 11938 | ATPase, Ca++ transporting, cardiac muscle, slow twitch 2 | -0,178 | 0,181 |
| Thap12 | 72981 | THAP domain containing 12 | 0,186 | -0,144 |
| Mfap3l | 71306 | Microfibrillar-associated protein 3-like | 0,196 | -0,195 |
| Cilk1 | 56542 | Ciliogenesis associated kinase 1 | 0,241 | -0,212 |
| Cep83os | 67723 | Centrosomal protein 83, opposite strand | 0,258 | -0,297 |
| Rft1 | 328370 | RFT1 homolog | 0,291 | -0,284 |
| Zfp949 | 71640 | Zinc finger protein 949 | 0,318 | -0,181 |
| Atp11b | 76295 | ATPase, class VI, type 11B | 0,326 | -0,225 |
| C430042M11Rik | 320021 | RIKEN cDNA C430042M11 gene | 0,392 | -0,339 |
| Spata6 | 67946 | Spermatogenesis associated 6 | 0,394 | -0,396 |
| Gca | 227960 | Grancalcin | 0,412 | -0,256 |
| Bcl2 | 12043 | B cell leukemia/lymphoma 2 | 0,422 | -0,381 |
| Gm6245 | 621629 | Predicted gene 6245 | 0,424 | -0,350 |
| Btc | 12223 | Betacellulin, epidermal growth factor family member | 0,498 | -0,365 |
| Rell1 | 100532 | RELT-like 1 | 0,533 | -0,469 |
| H2-K1 | 14972 | Histocompatibility 2, K1, K region | 0,542 | -0,230 |
| Gm14412 | 1E+08 | Predicted gene 14412 | 0,557 | -0,582 |
| Rimbp3 | 239731 | RIMS binding protein 3 | 0,574 | -0,295 |
| Gm10742 | 1E+08 | Predicted gene 10742 | 0,580 | -0,264 |
| Zfand4 | 67492 | Zinc finger, AN1-type domain 4 | 0,640 | -0,587 |
| Ptpn20 | 19256 | Protein tyrosine phosphatase, non-receptor type 20 | 0,843 | -1,184 |
| Clic6 | 209195 | Chloride intracellular channel 6 | 0,900 | -0,326 |
| Slc29a4 | 243328 | Solute carrier family 29 (nucleoside transporters), member 4 | 0,932 | -0,650 |
| Tec | 21682 | Tec protein tyrosine kinase | 1,356 | -0,471 |
| Irf6 | 54139 | Interferon regulatory factor 6 | 1,590 | -0,614 |
| Ly75 | 17076 | Lymphocyte antigen 75 | 2,102 | -0,515 |
| Il13ra2 | 16165 | Interleukin 13 receptor, alpha 2 | 3,116 | -0,881 |
| Krt5 | 110308 | Keratin-5 | 3,788 | -4,557 |
| Dsp | 109620 | Desmoplakin | 3,893 | -4,574 |
| Dsc3 | 13507 | Desmocollin-3 | 5,302 | -5,845 |

**Supplementary Table 1**: List of DEGs in the aged retina compared to the young retina rescued by NACET treatment. The log2 FC is indicated.
