# Supplementary material for: N-acetyl-l-cysteine ethyl ester (NACET) induces the transcription factor NRF2 and prevents retinal aging and diabetic retinopathy
Source: Redox Biol. 2025 Nov 3;88:103914. doi: 10.1016/j.redox.2025.103914 (PMC12793733; doi:10.1016/j.redox.2025.103914)
Supplement: Multimedia component 19 [file mmc19.docx]

|  | **FORWARD** | **REVERSE** | **Tm** |
| --- | --- | --- | --- |
| **NQO1** | CGCAGACCTTGTGATATTCCAG | CGTTTCTTCCATCCTTCCAGG | 57°C |
| **HMOX1** | TGACCCATGACACCAAGGAC | AGTGTAAGGACCCATCGGAGA | 58°C |
| **MGST1** | TCGTGACAAAGCAAATTGTCTGG | CCATTACCTGGGTGAGGTCAA | 63°C |
| **GSR** | GCCTTCACGAGTGATCCCAA | CTGCACCAACAATGACGCTG | 55°C |
| **GCLM** | GGAACCTGCTGAACTGGGG | CCCTGACCAAATCTGGGTTGA | 58°C |
| **GCLC** | GTTCTTGAAACTCTGCAAGAGAAG | CCTTCAATCATGTAACTCC | 55°C |
| **TXN** | TGGTGAAGCAGATCGAGAGC | ACATCCTGACAGTCATCCACAT | 57°C |
| **SLC7A11** | TCCCTACTATGGTCAGAAAG | TTGGGCAGATTGCCAAGATC | 57°C |
| **NEF2L2** | GCGCAGACATTCCCGTTTG | GACTGGGCTCTCGATGTGAC | 56°C |
| **GAPDH** | GAAGGTGAAGGTCGGAGTC | GAAGATGGTGATGGGATTTC | 57°C |

Supplementary Table 2. Primer couples used for the RT-qPCR.
