# Supplementary material for: N-acetyl-l-cysteine ethyl ester (NACET) induces the transcription factor NRF2 and prevents retinal aging and diabetic retinopathy
Source: Redox Biol. 2025 Nov 3;88:103914. doi: 10.1016/j.redox.2025.103914 (PMC12793733; doi:10.1016/j.redox.2025.103914)
Supplement: Multimedia component 20 [file mmc20.docx]

| **Antigene** | **Antibody** | **Source/Type** | **Cat #** | **Working dilution** |
| --- | --- | --- | --- | --- |
| **NRF2** | NRF2 (D1Z9C) XP | Rabbit mAb | #12721 - Cell Signaling | 1:2000 |
| **NRF2** |  | Rabbit pAb | 16396-1-AP - Proteintech | 1:2000 |
| **HMOX1** |  | Rabbit pAb | # ab13243 - Abcam | 1: 1000 |
| **KEAP1** | KEAP1(OTI1B4-formerly 1B4) | Mouse mAb | #TA502059 - Origene | 1:1000 |
| **NQO1** | NQO1 Antibody (A180) | Mouse mAb | # sc-32793 - Santa Cruz Biotechnology | 1:5000 |
| **NQO1** | NQO1 Antibody (EPR3309) | Rabbit mAb | # ab80588 - Abcam | 1:5000 |
| **IL-1β** |  | Rabbit pAb | #P420B – ThermoFischer Scientific | 1:200 |
| **IL-6** | IL-6 Antibody (10E5) | Mouse mAb | # sc-57315 - Santa Cruz Biotechnology | 1: 200 |
| **GFAP** | GFAP antibody (EPR19996) | Rabbit mAb | # ab80588 - Abcam | 1 :5000 |
| **GAPDH** | Anti-GAPDH (7E4-H6-H6) | Mouse mAb-IgM | #STJ99066 - St John’s Laboratory | 1:5000 |
| **β -ACTIN** | Anti-β-Actin (AC-74) | Mouse mAb | #A2228 - Sigma-Aldrich | 1:2500 |
| **Secondary Anti-Rabbit** | Peroxidase-conjugated AffinityPure Goat Anti-Rabbit IgG (H+L) | Goat polyclonal | #111-035-003 - Jackson Immuno Research | 1:5000 |
| **Secondary Anti-Rabbit** | Goat Anti-Rabbit IgG (H + L)-HRP Conjugate | Goat pAb | #1706515 – Bio-Rad Laboratories | 1:5000 |
| **Secondary Anti-Mouse** | Peroxidase-conjugated AffinityPure Goat Anti-Mouse IgG (H+L) | Goat polyclonal | #115-035-003 - Jackson Immuno Research | 1:5000 |
| **Secondary Anti-Mouse** | Peroxidase-conjugated AffinityPure Goat Anti-Mouse IgG+IgM (H+L) | Goat polyclonal | #115-035-044 - Jackson Immuno Research | 1:5000 |
| **Secondary Anti-Mouse** | Rabbit Anti-Mouse IgG (H + L)-HRP Conjugate | Rabbit pAb | #A9044 - Sigma-Aldrich-Merck | 1:5000 |

Supplementary Table 3. Primary and secondary antibodies used in Western blot.
