# Supplementary material for: N-acetyl-l-cysteine ethyl ester (NACET) induces the transcription factor NRF2 and prevents retinal aging and diabetic retinopathy
Source: Redox Biol. 2025 Nov 3;88:103914. doi: 10.1016/j.redox.2025.103914 (PMC12793733; doi:10.1016/j.redox.2025.103914)

**Figure 2a – NRF2 expression**


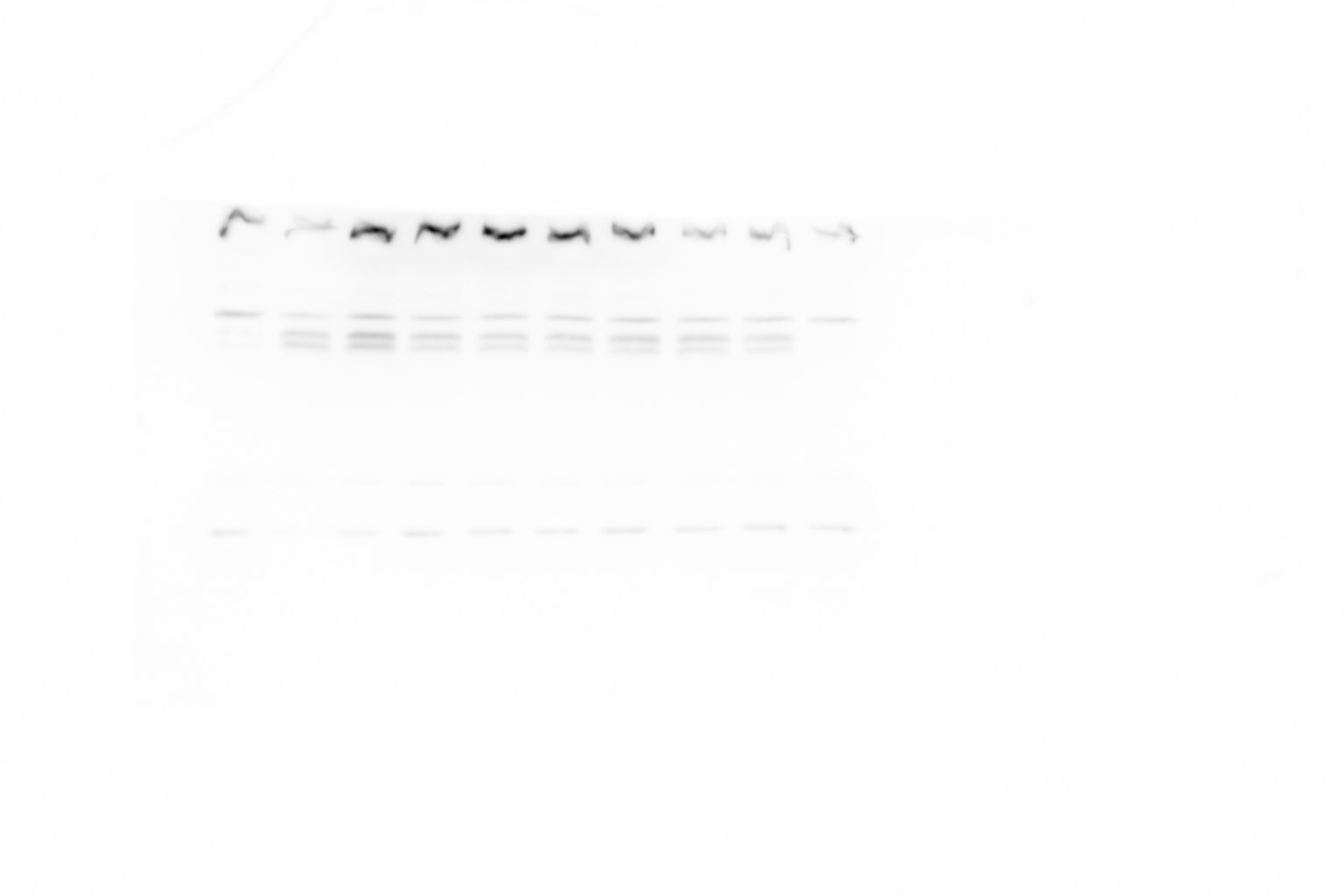

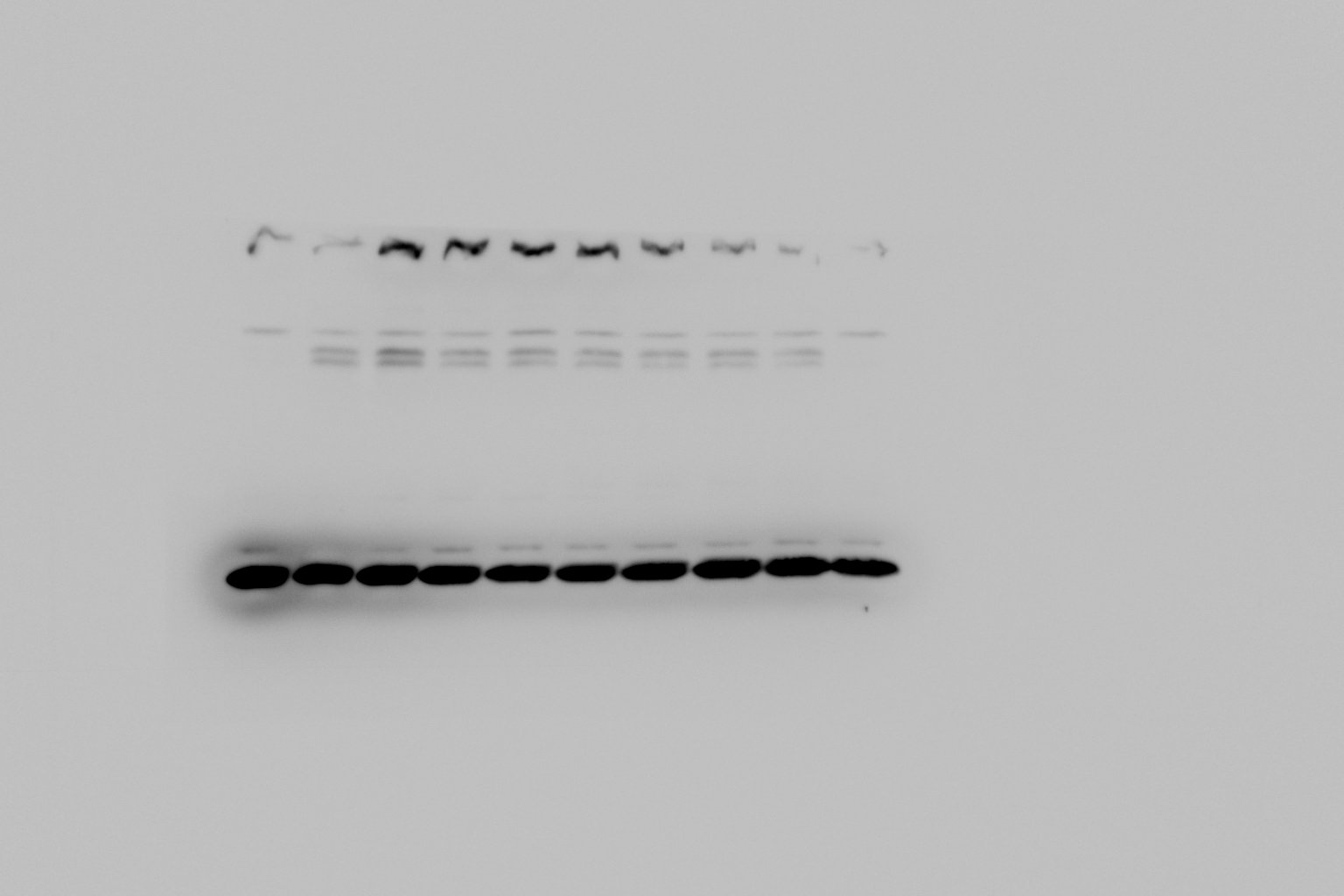


Figure 2a

IB: anti-GAPDH (over IB anti-NRF2)

Figure 2a

IB: anti-NRF2


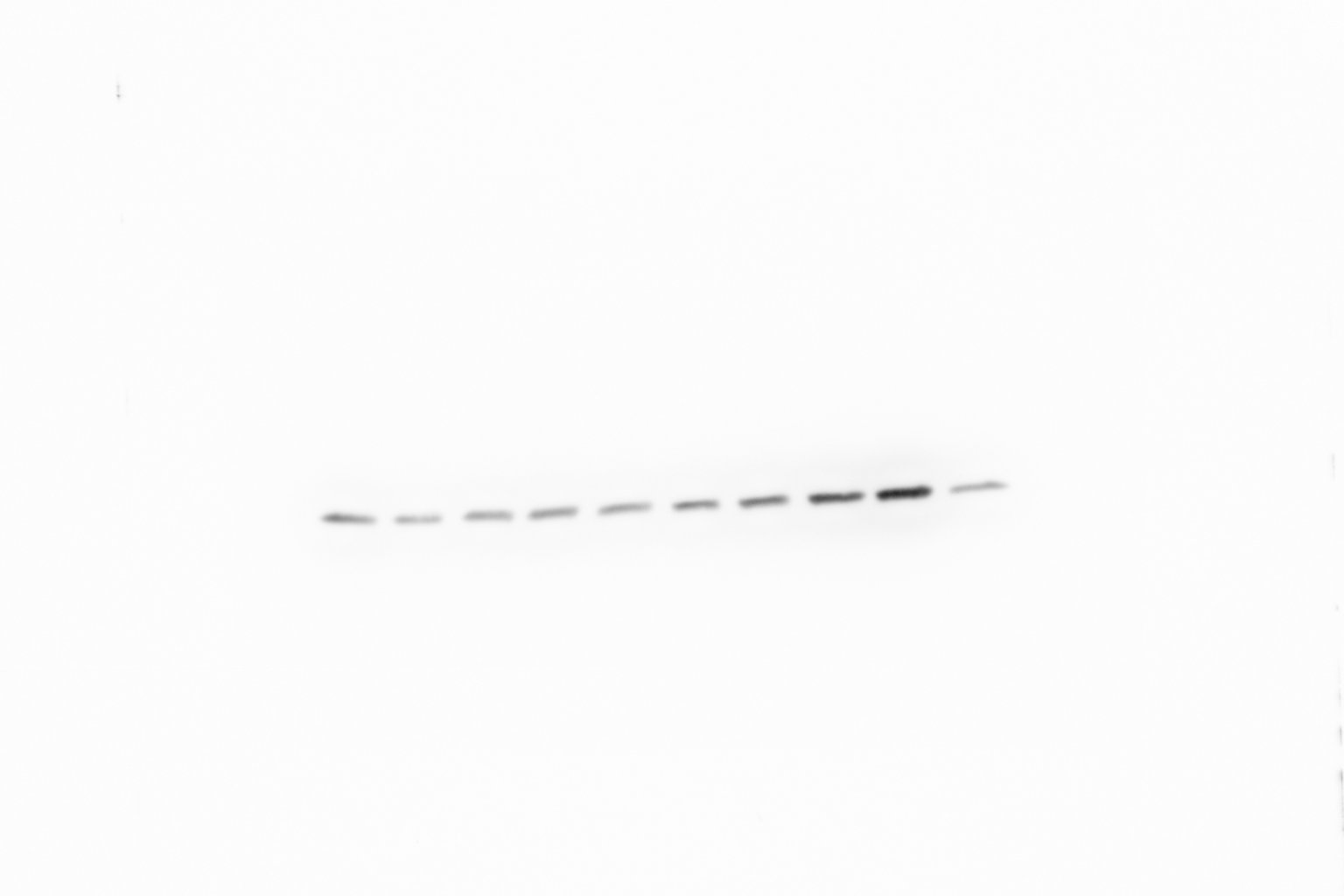

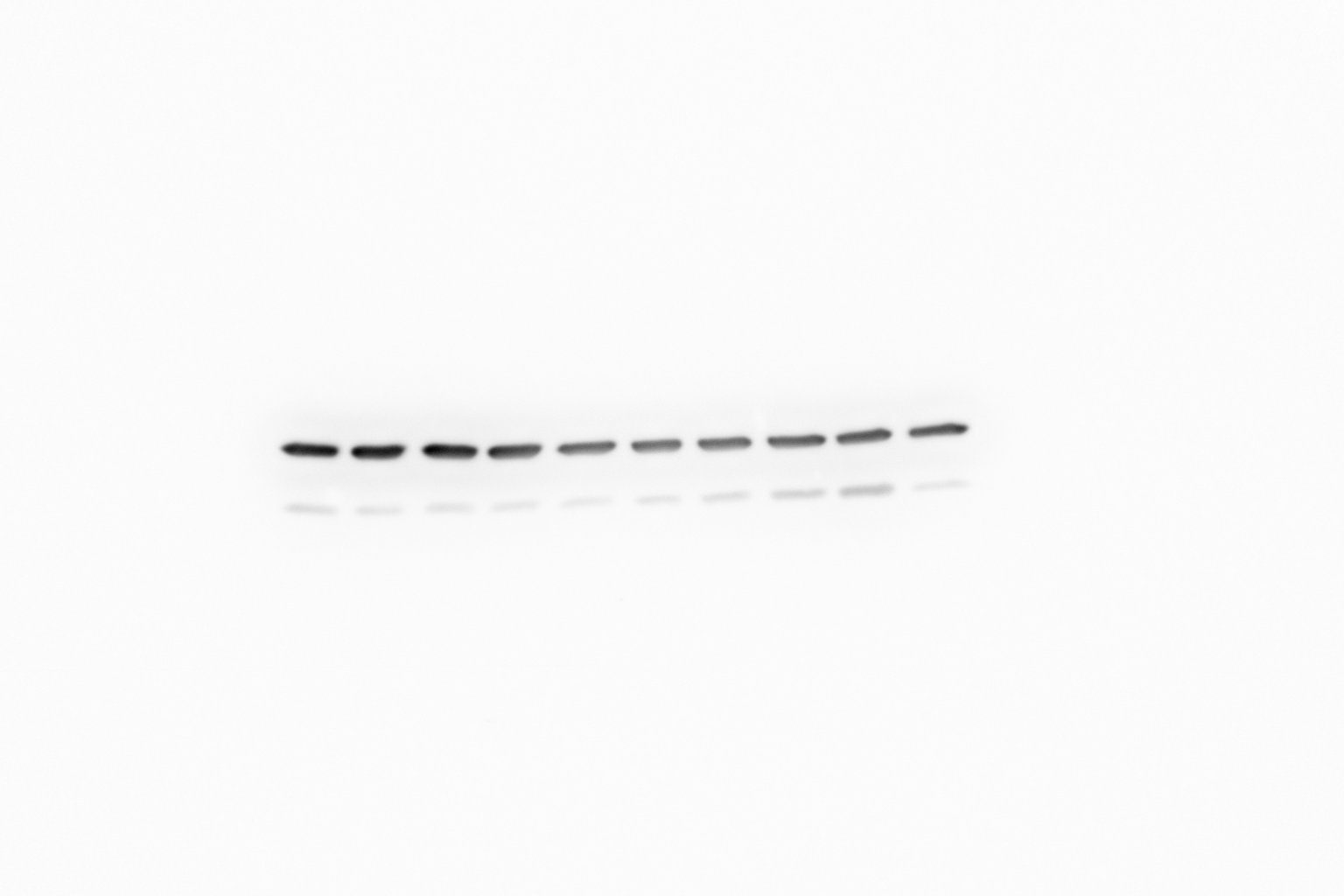


Figure 2a

IB: anti-NQO1

Figure 2a

IB: anti-GAPDH (over IB anti-NQO1)

**Figure 2b– NRF2 expression**


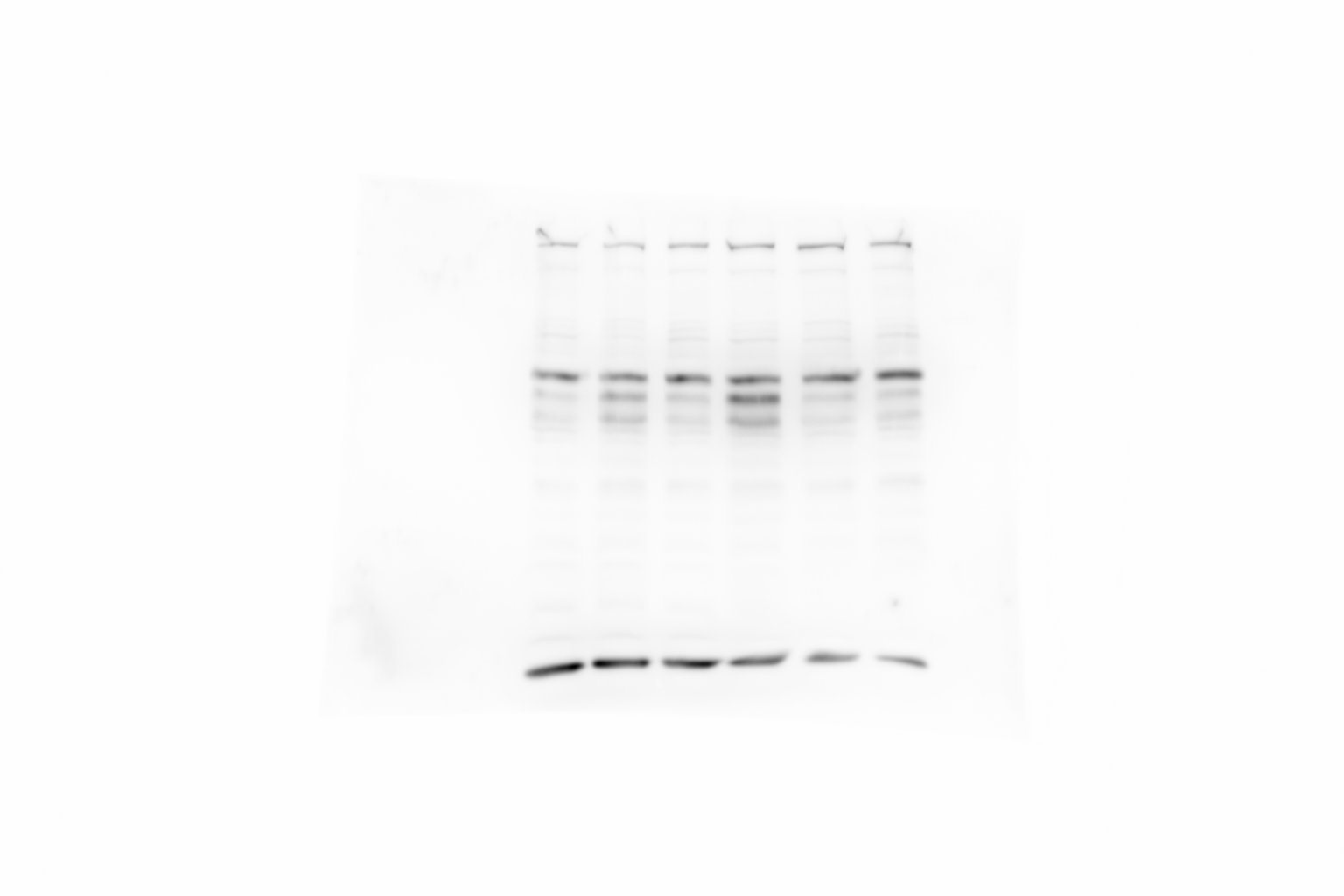


Figure 2b

IB: anti-NRF2


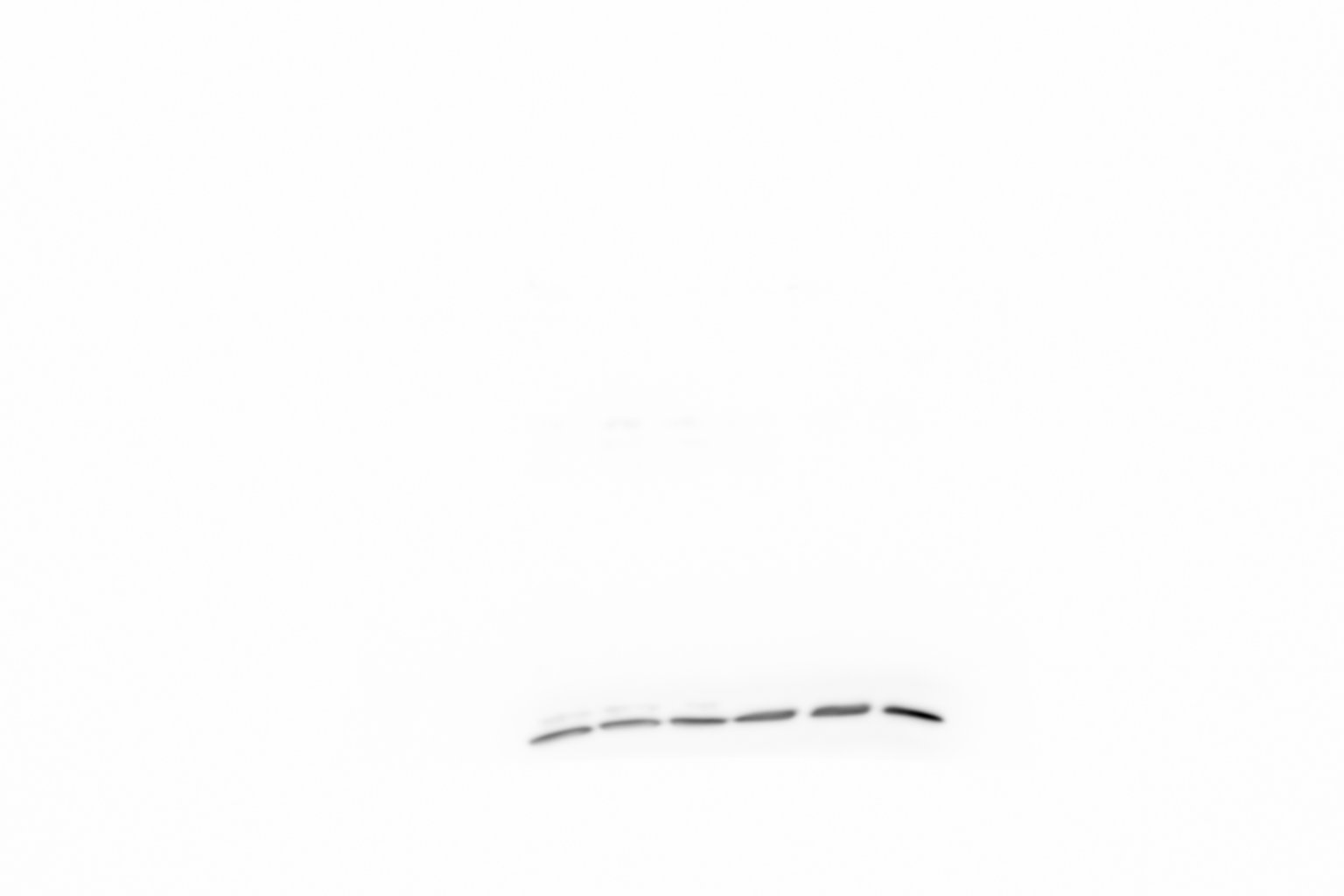


Figure 2b

IB: anti-GAPDH (over IB anti-NRF2)

**Figure 2b– NRF2 expression replicates**

**
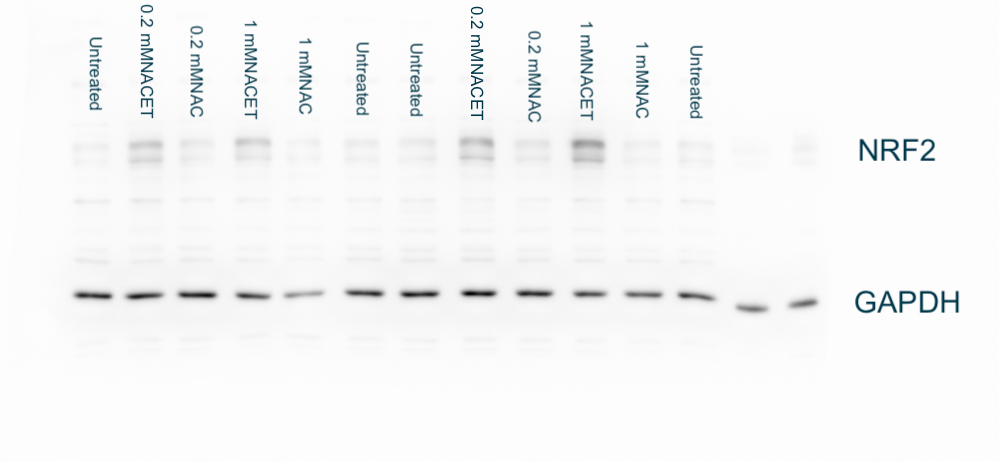
**

**Figure 2c – NRF2 expression**


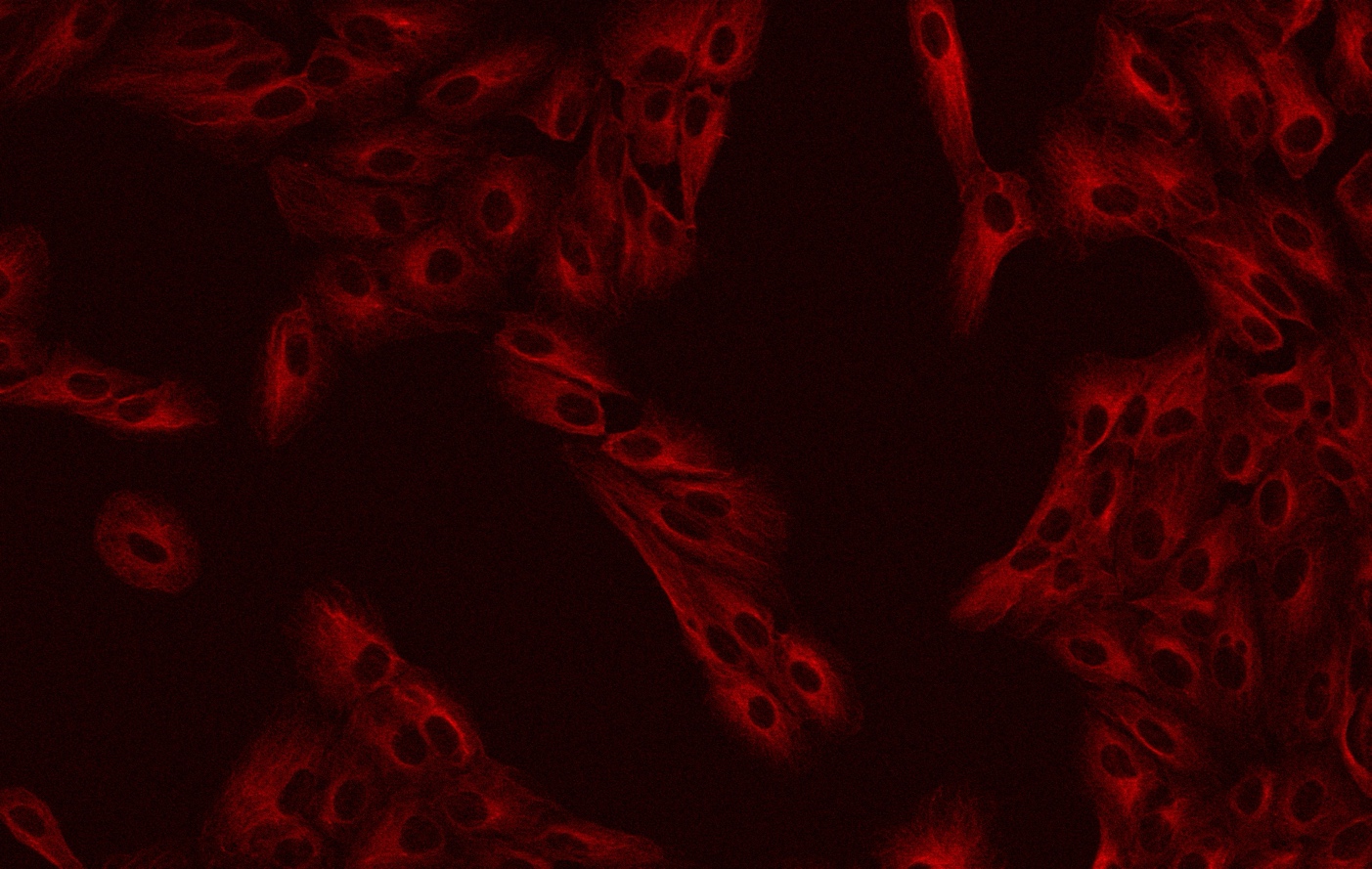

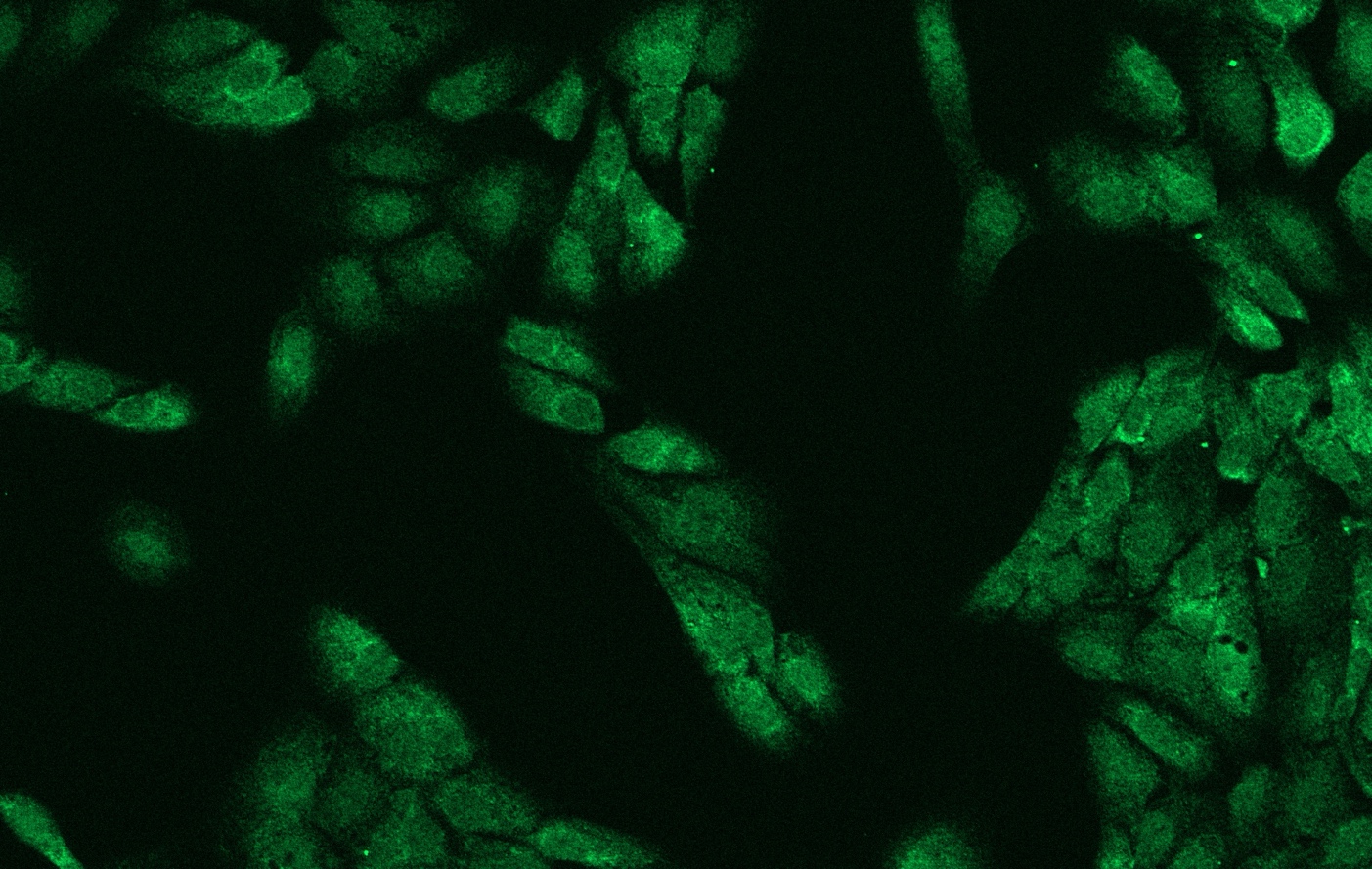

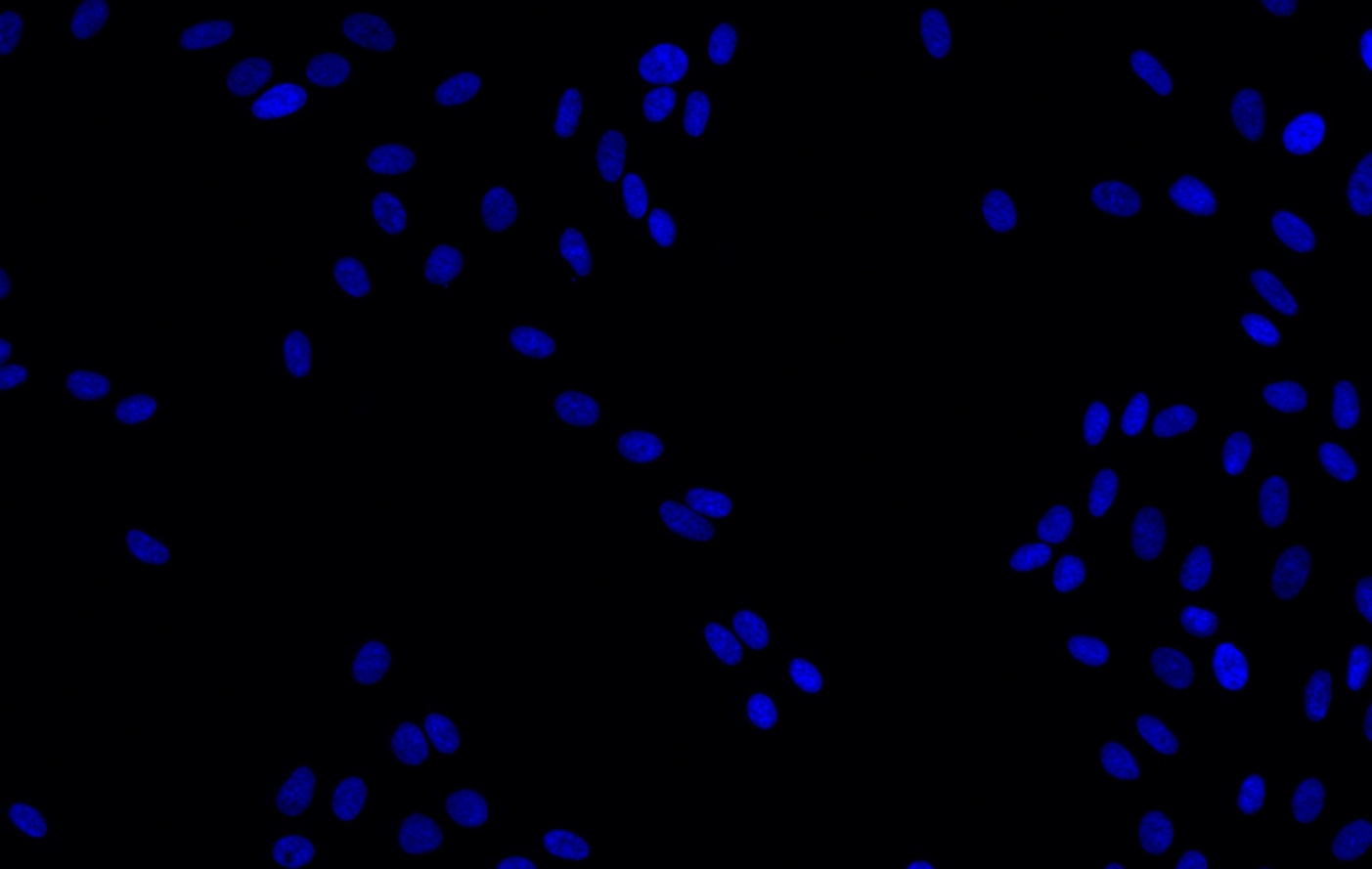

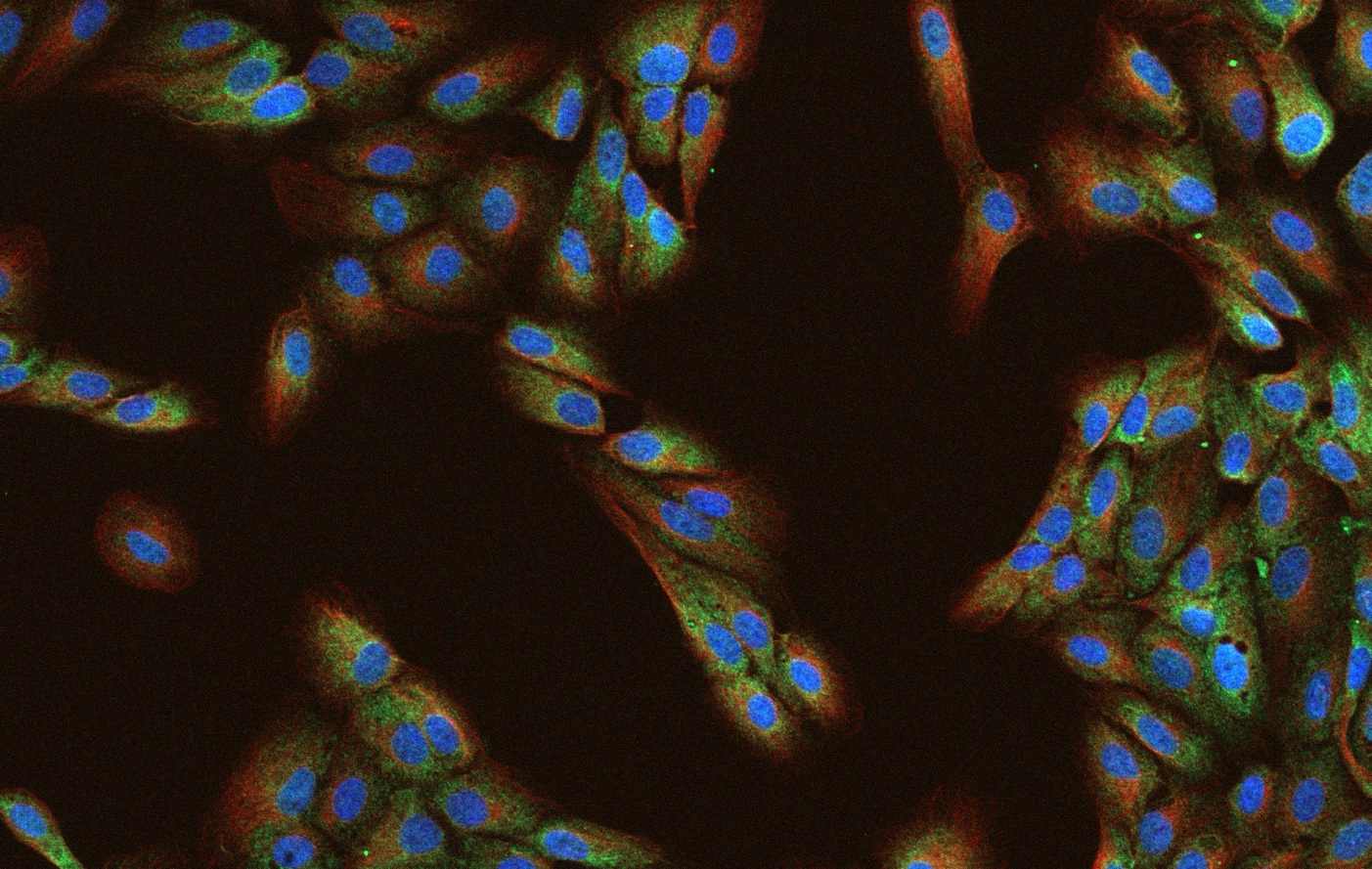


Figure 2C

Green (NRF2) 6h

Figure 2C

Red (Tubulin) 6h

Figure 2C

DAPI 6h

Figure 2C

Merge 6h


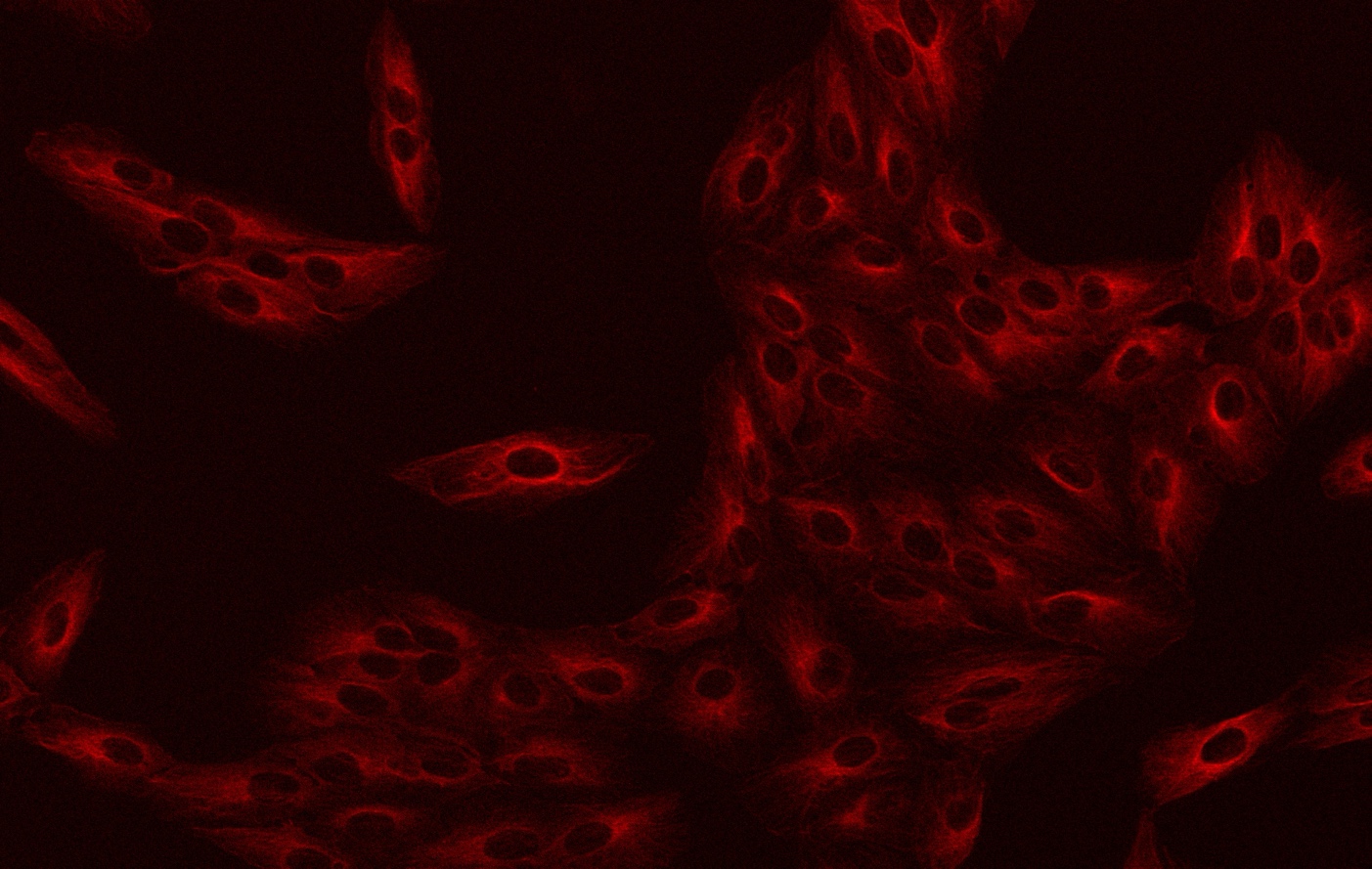

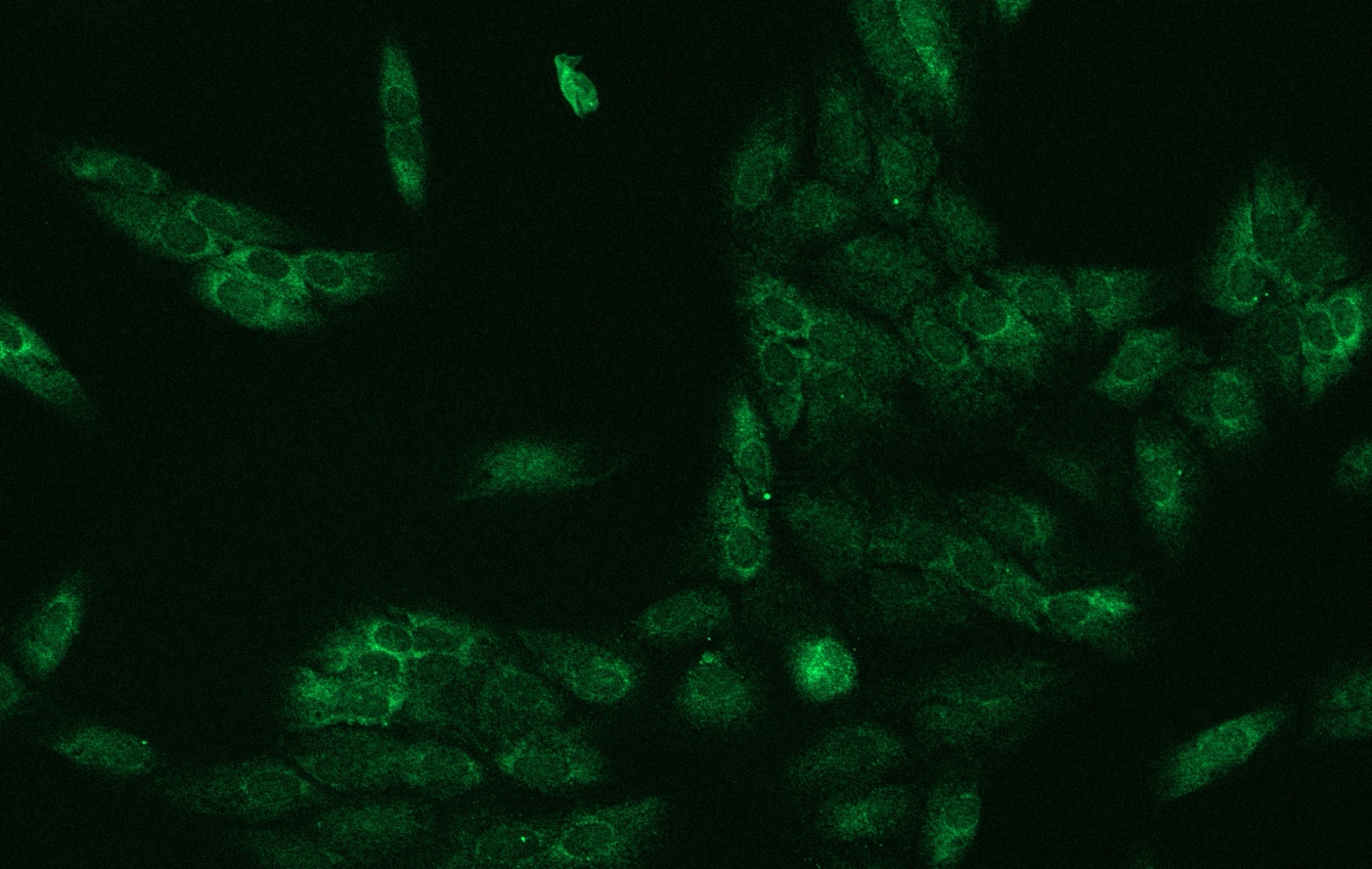

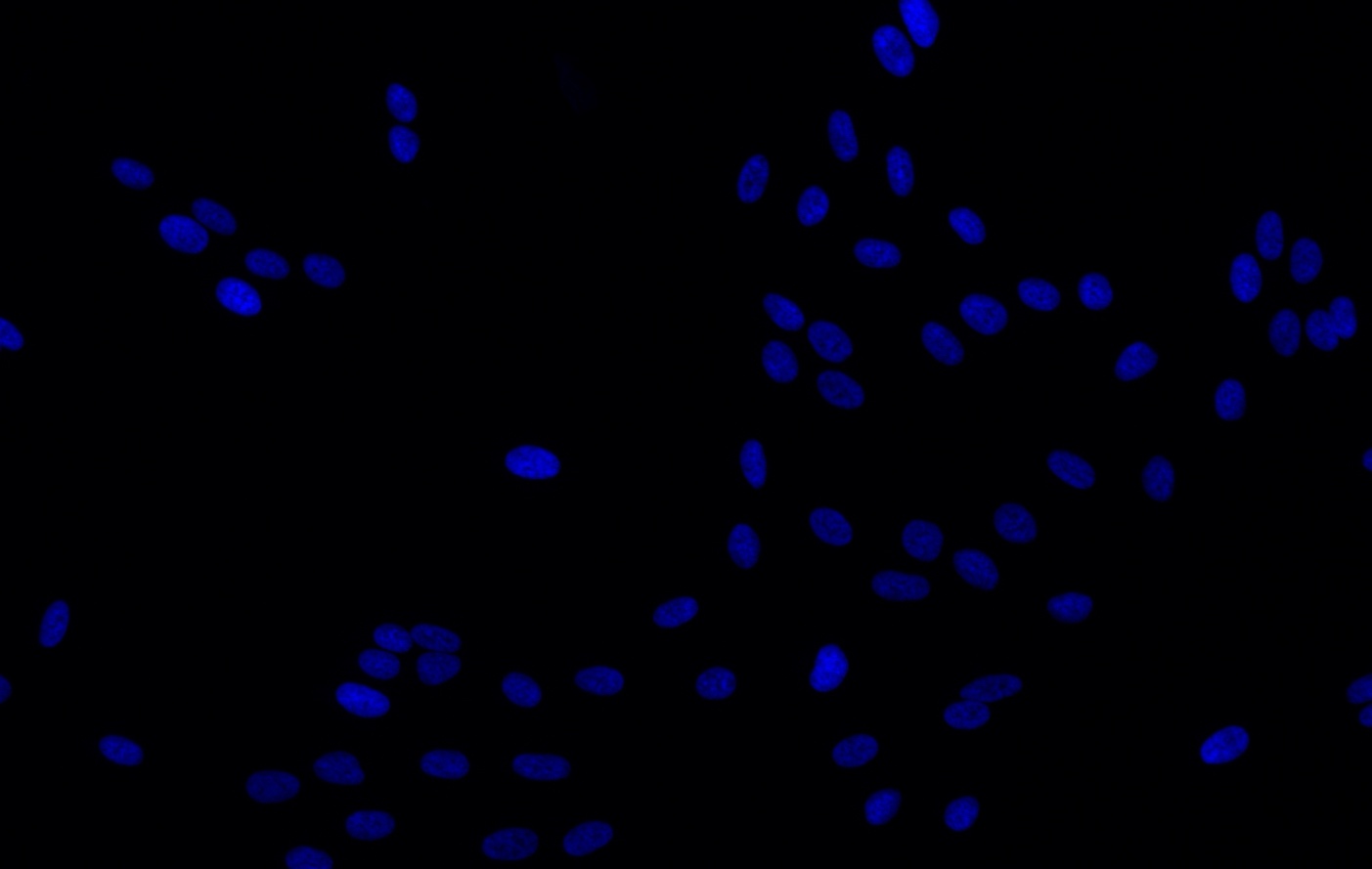

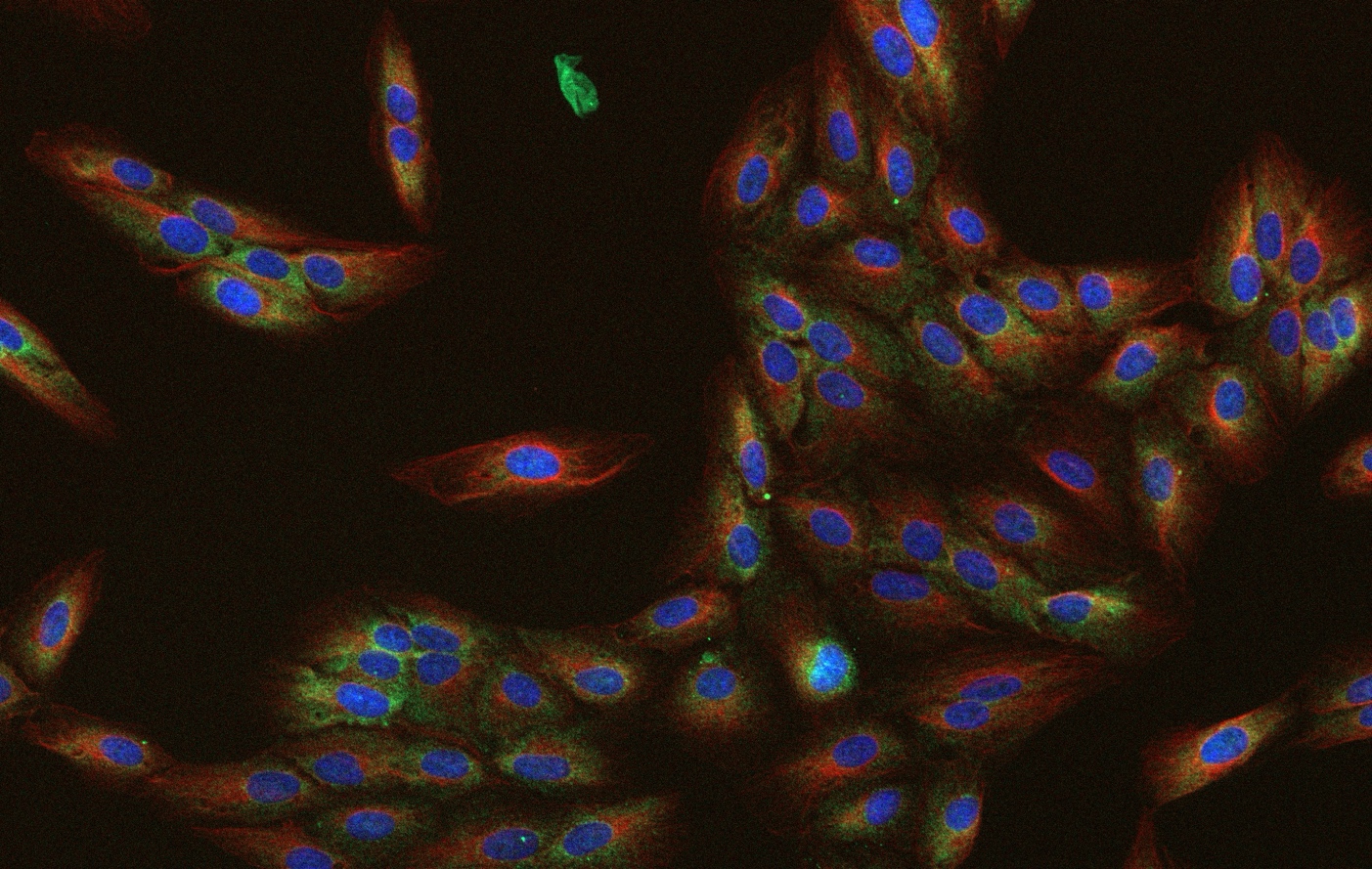


Figure 2C

Green (NRF2) ctrl

Figure 2C

Red (Tubulin) ctrl

Figure 2C

DAPI ctrl

Figure 2C

Merge ctrl

**Figure 3c – NRF2 expression**


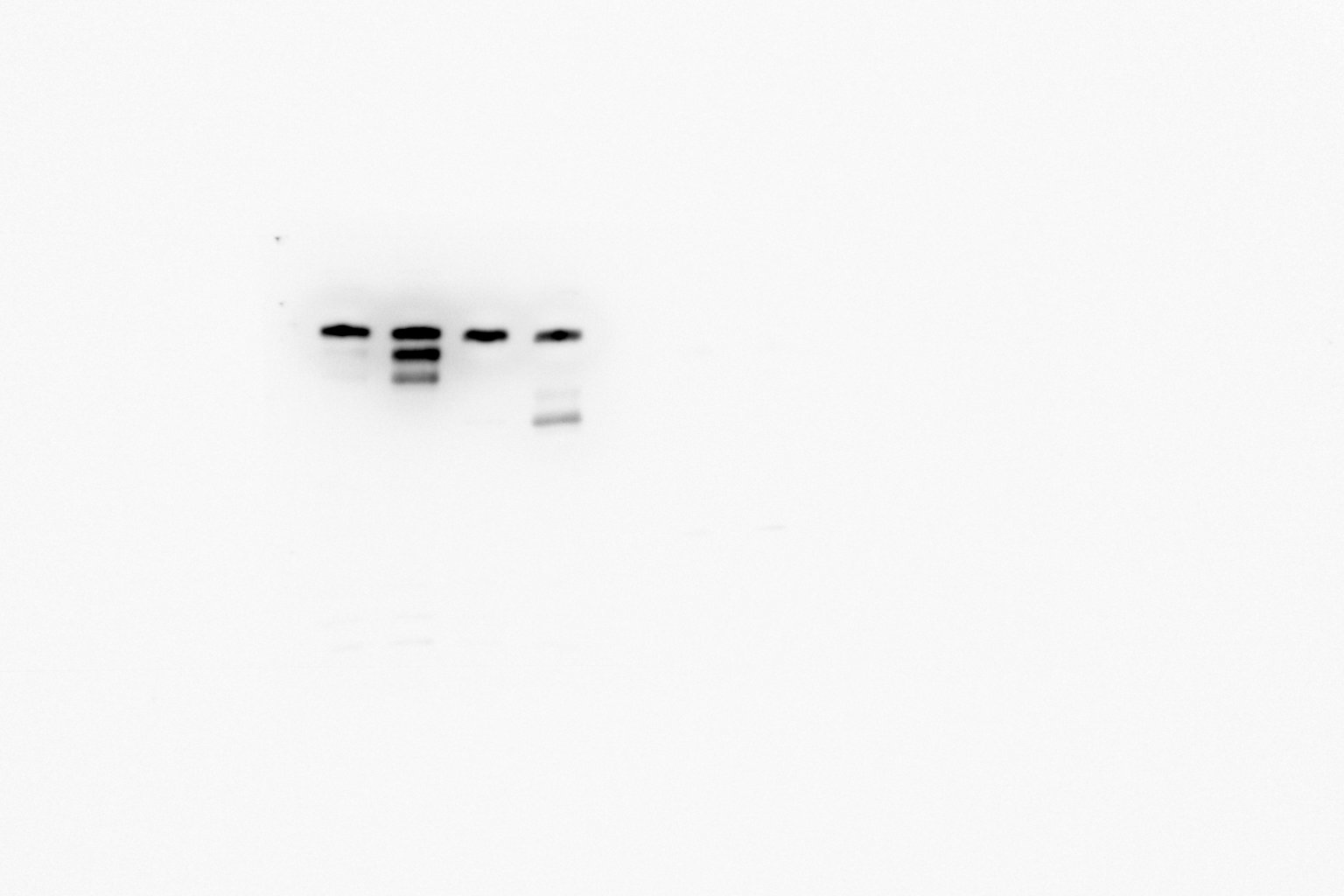

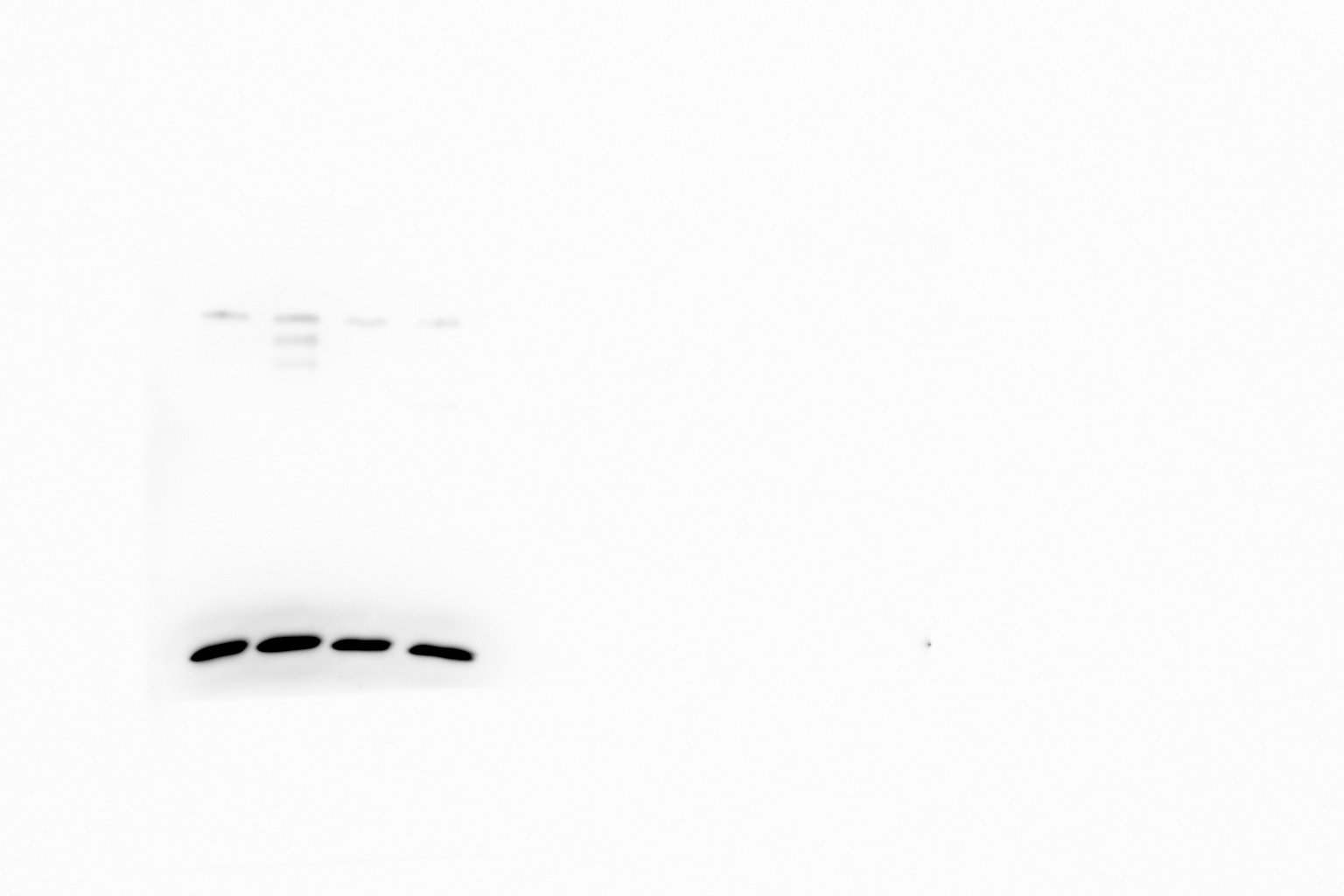


Figure 3c

IB: anti-GAPDH (over IB anti-NRF2)

Figure 3c

IB: anti-NRF2

**Figure 8a-b (Oxidative stress markers)**


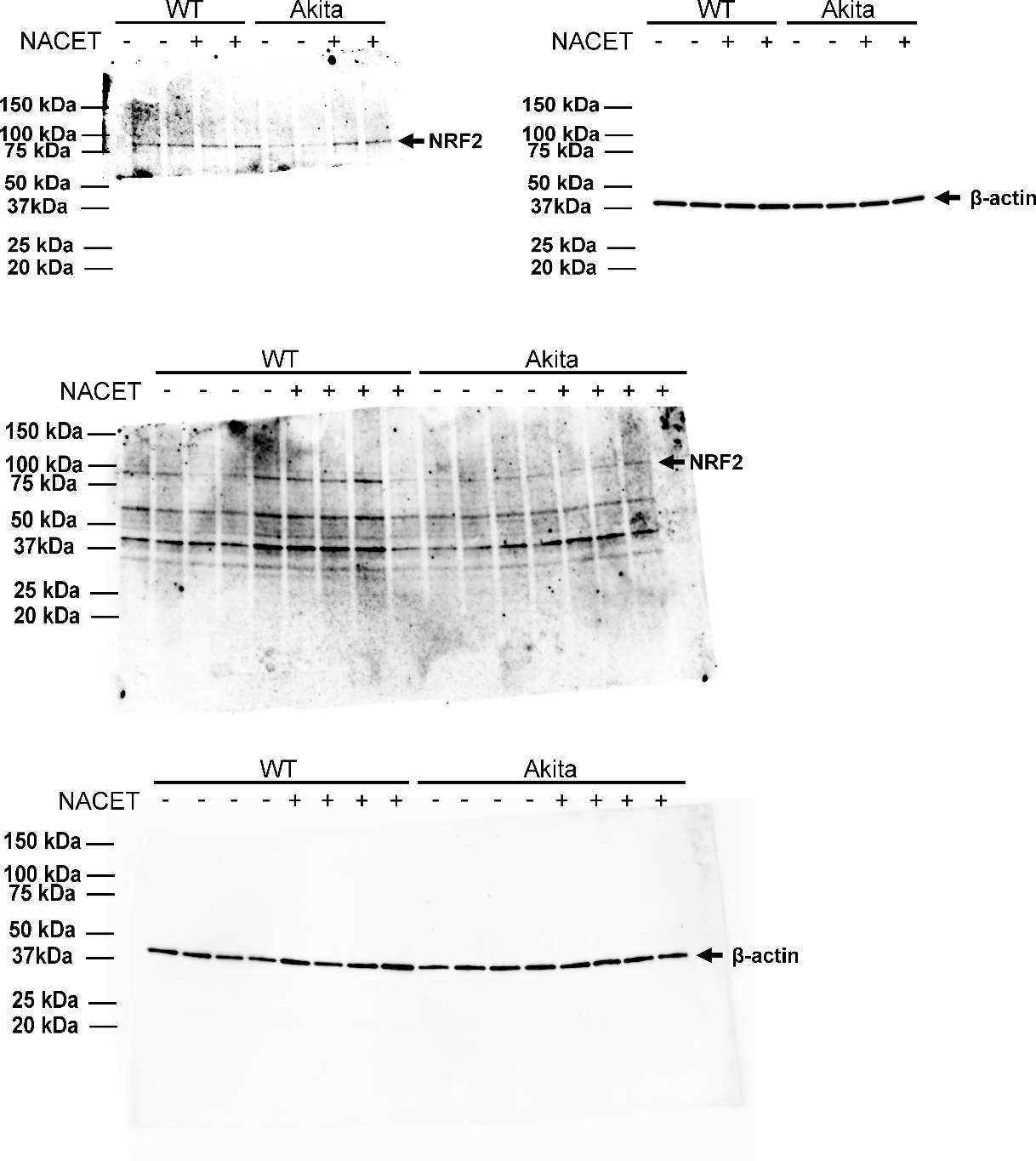


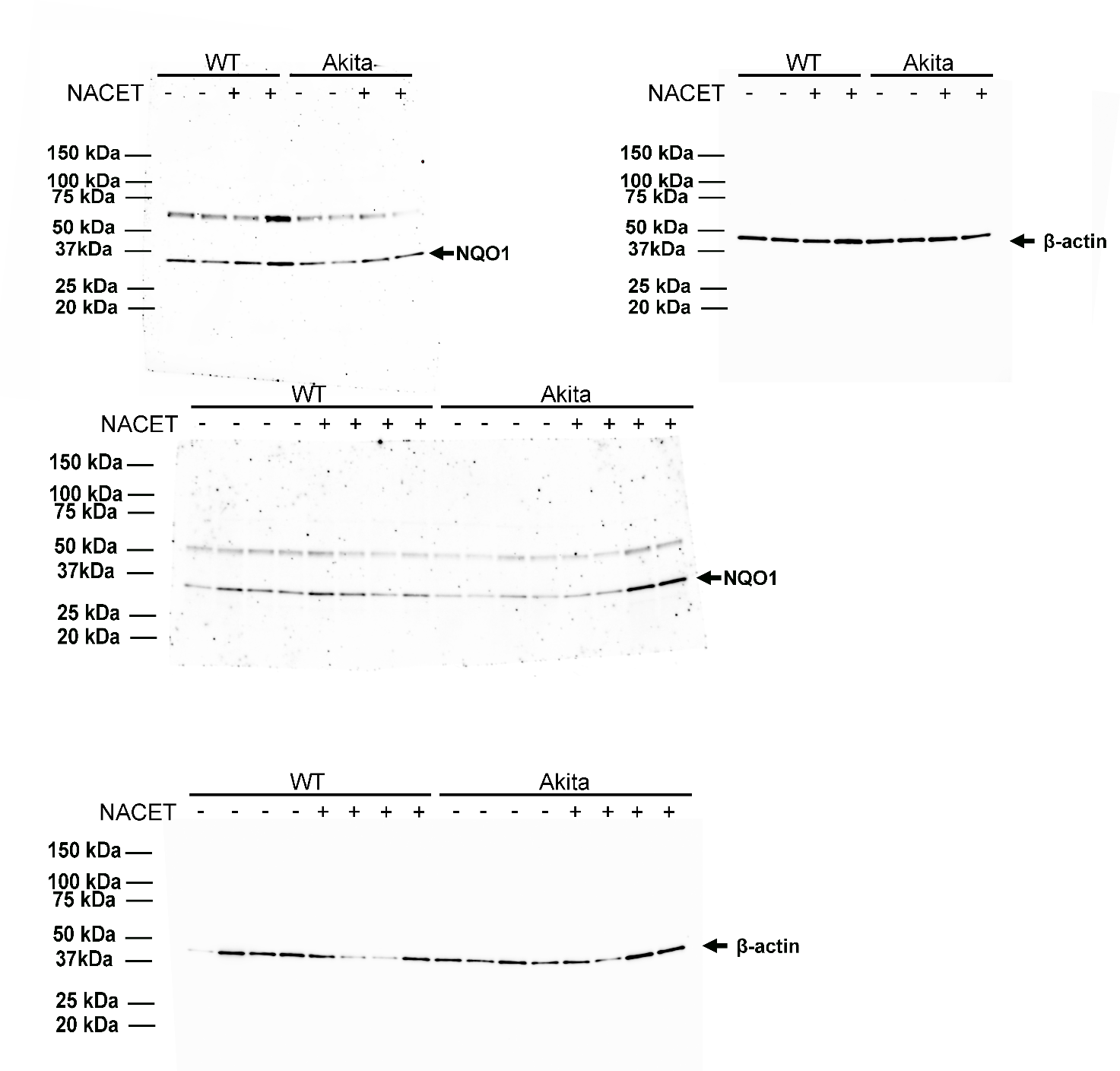


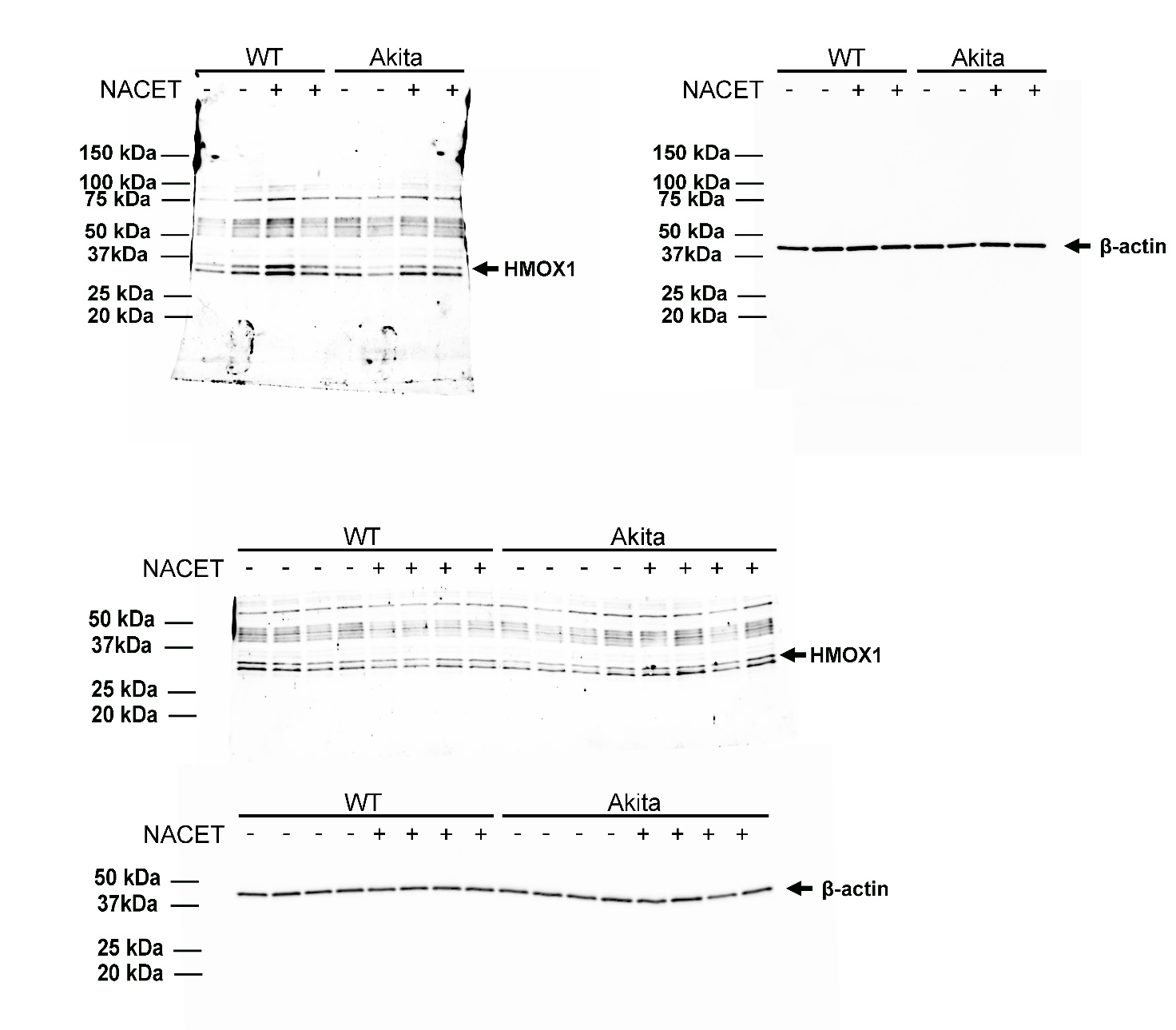


**Figure 8c-d (Inflammatory markers)**


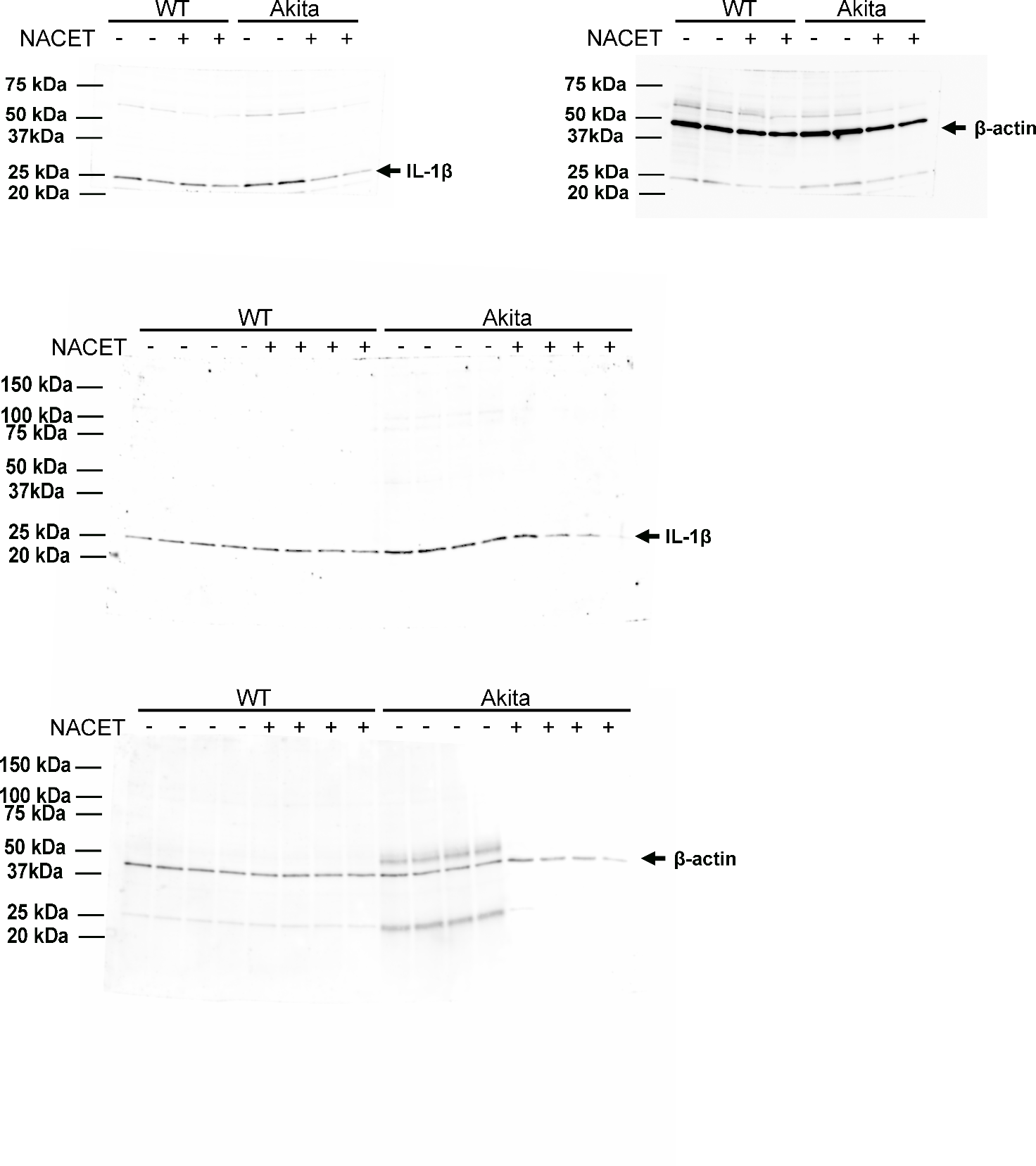


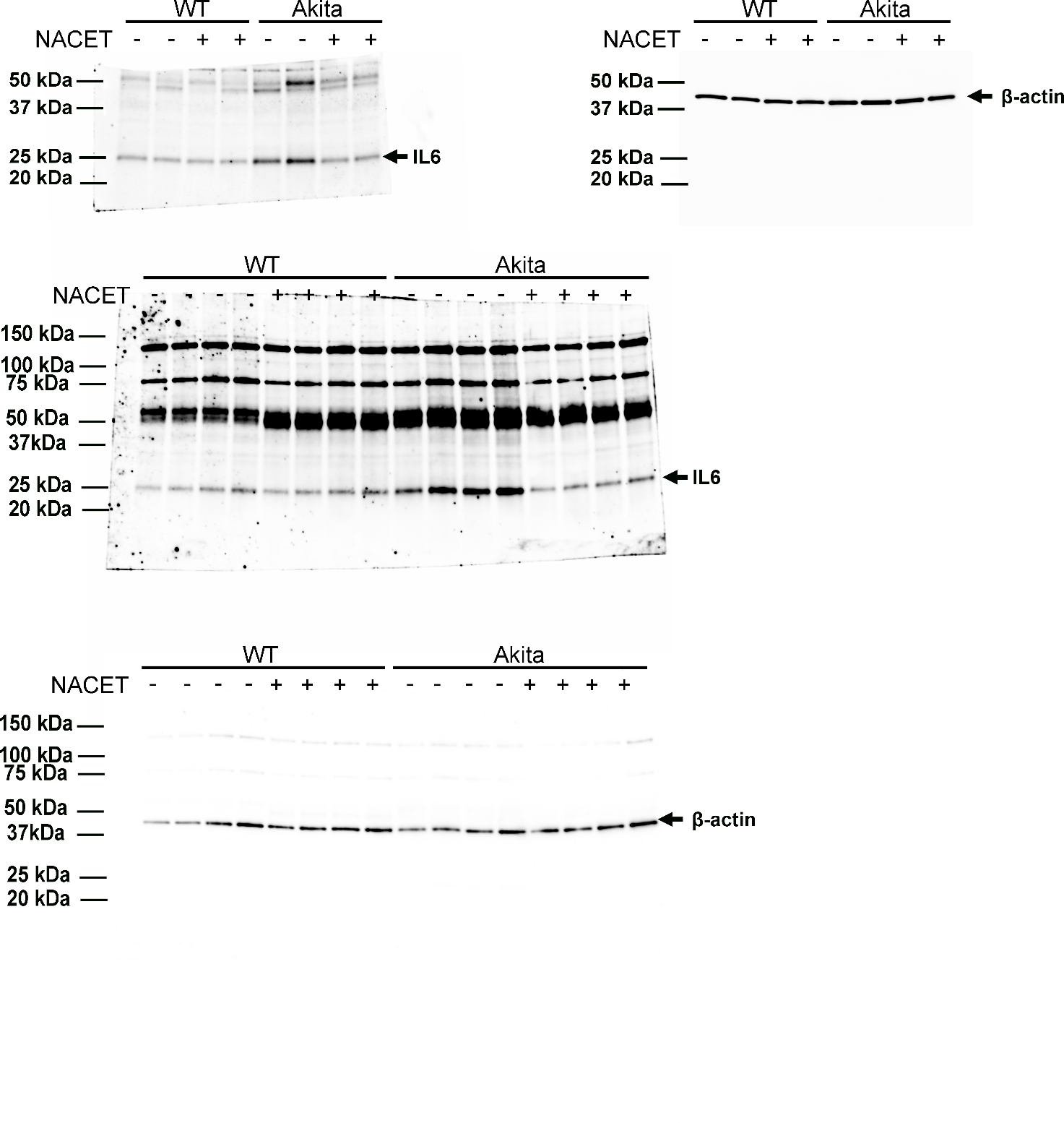


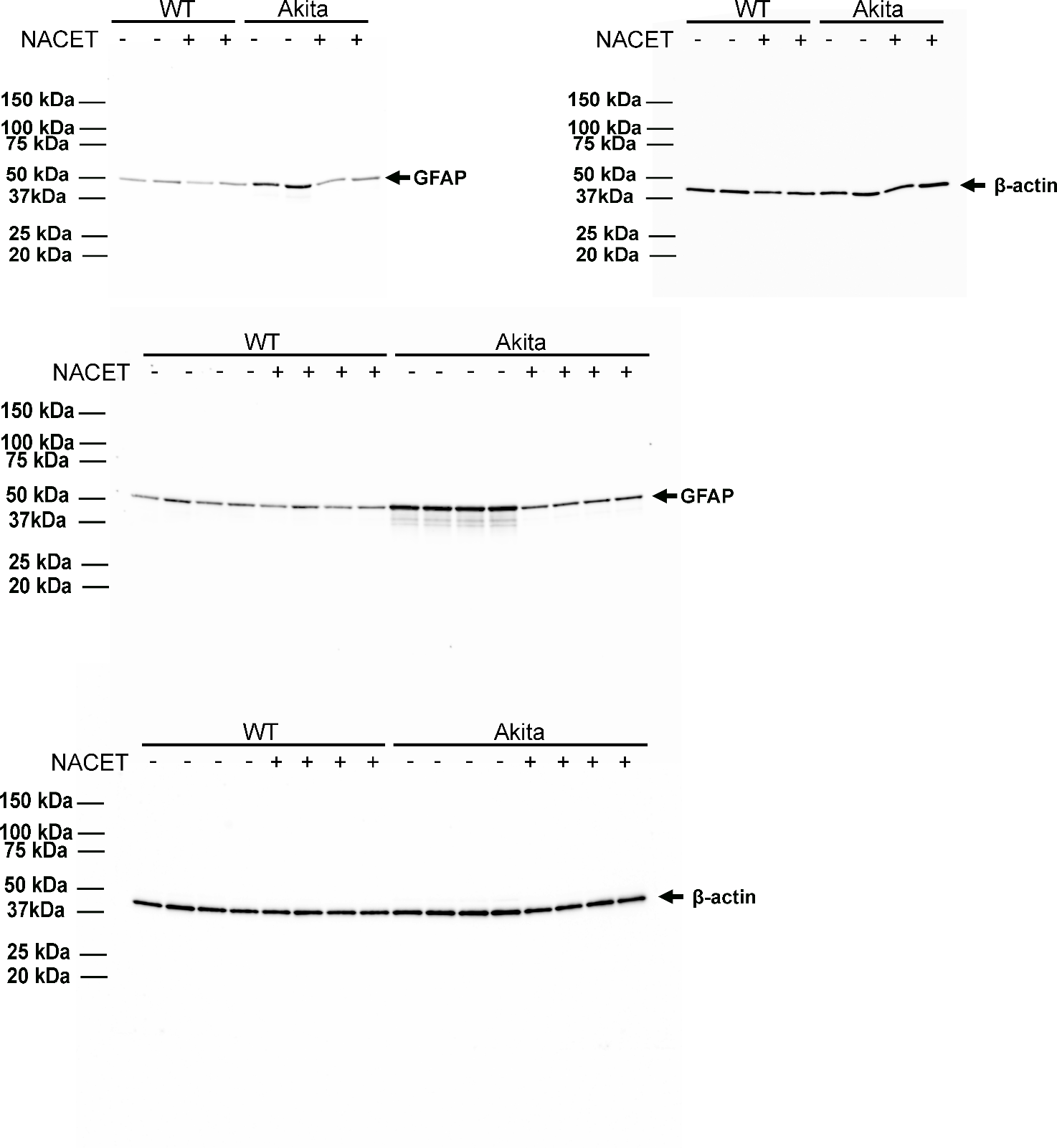

Supplement: Multimedia component 21 [file mmc21.docx]
